# The effects of varying levels of trace mineral supplementation on performance, carcass characteristics, mineral balance, and antibody concentrations in feedlot cattle

**DOI:** 10.1093/tas/txac093

**Published:** 2022-07-28

**Authors:** Brittany A Lippy, Colton A Robison, Blake K Wilson

**Affiliations:** Department of Animal and Food Sciences, Oklahoma State University, Stillwater, OK 74074, USA; Department of Animal and Food Sciences, Oklahoma State University, Stillwater, OK 74074, USA; Department of Animal and Food Sciences, Oklahoma State University, Stillwater, OK 74074, USA

**Keywords:** copper, feedlot nutrition, requirements, supplementation, zinc

## Abstract

The objective of this experiment was to determine the effects of increasing trace mineral (TM) supplementation on finishing cattle performance, carcass characteristics, TM balance, and antibody concentrations. Commercial Angus steers (*n* = 240; body weight, BW = 291 kg ± 27.4) were stratified by arrival BW and source and randomly assigned to 1 of 4 experimental treatments in a randomized complete block design (12 pens/treatment; 5 steers/pen). All steers underwent a TM depletion period for a minimum of 42-d prior to the administration experimental treatments. Treatments included a negative control (CON) in which cattle received no additional TM supplementation or TM supplementation treatments in which cattle received added Co, Cu, I, Mn, Se, or Zn from inorganic TM sources at 2016 Nutrient Requirements of Beef Cattle (NASEM) requirement levels (1X), at 2 times NASEM requirements (2X), or at 4 times NASEM requirements (4X). Selenium was included at 0.1, 0.2, and 0.3 mg/kg for 1X, 2X, and 4X respectively, based on federal law. There was no difference in overall BW, average daily gain (ADG), dry matter intake (DMI), or gain to feed (G:F) due to TM supplementation (CON vs. SUPP *P* ≥ 0.47). There was no difference in hot carcass weight, rib eye area, fat thickness, dressing percentage, marbling score, or USDA Yield Grade due to TM supplementation (CON vs. SUPP *P* ≥ 0.30). One steer was chosen at random from each pen to be evaluated for serum and liver TM status and antibody concentrations to common respiratory viruses. There was a treatment × day interaction for serum Co and liver Cu and Se (*P* < 0.0001). Serum Co was greatest for the 4X treatment from d 28 through harvest. Liver Cu was greatest for the 2X and 4X treatments from d 56 through harvest. Liver Se was greatest for 2X and 4X from d 28 through harvest. Serum Zn was greatest for the 4X treatment (*P* = 0.02). There was an effect of day on liver Co, Fe, Mn, Mo, and Zn (*P* ≤ 0.0001) and serum Cu, Mn, Mo, Se, and Zn (*P* ≤ 0.002). Individual TM concentrations differed over time; however, none were ever considered deficient or toxic based on published reference ranges. There was an effect of time on bovine viral diarrhea virus Type 1A, bovine herpesvirus type 1, and bovine parainfluenza 3 virus antibody concentrations (*P* ≤ 0.0001). Supplementation of TM above NASEM requirements did not affect overall cattle performance, carcass characteristics, or antibody concentrations, but did affect the storage and circulation of certain TM.

## INTRODUCTION

Combined and individual trace mineral (TM) supplementation of Cobalt (Co), Copper (Cu), Iron (Fe), Iodine (I), Manganese (Mn), Selenium (Se), and Zinc (Zn) has produced variable results in feedlot cattle. Research indicates that supplying the 7 TM with published requirements does not consistently enhance performance or carcass characteristics (Downer et al., 1981; Engle and Spears, 2000, 2001; Legleiter et al., 2005; Malcolm-Callis et al., 2000; Tiffany et al., 2003). Typically, feedlot diets are fortified with TM supplements due to perceptions of insufficient and variable TM content of feedstuffs, the presence of antagonistic compounds, and unknown cattle TM status upon feedlot arrival. The current NASEM (2016) recommendations for TM dietary inclusion, are as follows: 15.0 mg Co/kg, 10.0 mg Cu/kg, 50.0 mg Fe/kg, 0.50 mg I/kg, 20.0 mg Mn/kg, 0.10 mg Se/kg, and 30.0 mg Zn/kg (DM basis) to meet the requirements of growing and finishing cattle. These requirements consider TM provided by the entire diet. However, professionals within the industry commonly do not take basal diet TM concentrations into consideration. From a 2015 survey, consulting nutritionists recommend on average: 0.82 mg Co/kg, 17.0 mg/kg of Cu, 13.8 mg Fe/kg, 0.73 mg I/kg, 47.9 mg Mn/kg, 0.24 mg Se/kg, and 87.3 mg Zn/kg (DM basis) of supplemental TM be added to the basal diet (Samuleson et al., 2016). These average recommendations of added TM are approximately 2 to 4 times the published requirements for total dietary TM. Much published TM research lacks an unsupplemented negative control, dietary concentrations similar to industry diets, and a depletion period to ensure TM status was equivalent before experimental treatment allocation. Therefore, the objectives of this experiment were to determine the effects of increasing concentrations of TM supplementation on 1) feedlot cattle performance and carcass characteristics, 2) serum and liver TM status, and 3) respiratory virus antibody concentrations. We hypothesized that TM supplementation at or above published TM requirements would result increased serum and liver TM concentrations, but similar performance and carcass characteristics.

## MATERIALS AND METHODS

All procedures were approved by the Institutional Animal Care and Use Committee at Oklahoma State University (Animal Care and Use Protocol number: AG-19-8)

### Cattle and Processing

This experiment used a total of 240 crossbred Angus steers that were received in 2 shipments from central South Dakota. While cattle were received 5 d apart, treatment allocation was staggered by 7 d to accommodate logistical challenges due to the time required to obtain body weights (BW) and biological samples and to keep sampling intervals consistent among groups.

In mid-February 2020, 164 crossbred Angus steers (BW = 297 ± 31.2 kg) were transported on 2 trucks approximately 948 km from Burke, SD to the Willard Sparks Beef Research Center (WSBRC) in Stillwater, OK. Of these initial 164 steers, 140 were initially processed upon arrival to be allocated to the experiment. The arrival processing procedures for the 140 steers included collection of individual BW and application of individual identification tags. Cattle were then held in a pen overnight with ad libitum access to prairie hay and water. The initial 140 processed cattle were deemed Group 1 (G1).

The remaining 24 head from the first shipment were placed in a feedlot pen and were fed ad libitum prairie hay and water until the arrival of the second shipment of cattle. Five days later, 81 additional steers (initial BW = 280 ± 19.1 kg) were transported on 1 truck approximately 1,328 km from Mobridge, South Dakota to the WSBRC. These 81 steers were combined with the remaining 24 cattle from the first shipment for processing and deemed Group 2 (G2). Five steers (the 3 steers with the heaviest arrival BW and the 2 steers with the lightest arrival BW) from G2 were removed prior to treatment allocation. The remaining 100 animals from G2 were individually weighed, tagged, and held in a pen overnight with ad libitum access to prairie hay and water.

Cattle from both groups (G1 *n* = 140; G2 *n* = 100) were processed the day following collection of arrival BW (this processing occurred either 41 or 43 d prior to initiation of experimental TM treatments on d 0 depending on group). Steers were blocked by BW within group and randomly allocated to pens within block. Processing procedures were similar to that described by Warner et al. (2020). Steers were individually weighed, implanted (Component TE-IS; Elanco Animal Health, Greenfield, IN), vaccinated against clostridial (Vision with SPUR; Merck Animal Health, Madison, NJ), and viral and bacterial respiratory pathogens (Nuplura PH and Titanium 5 + PH-M; Elanco Animal Health), administered an anthelmintic (Safeguard; Merck Animal Health), and a pour-on insecticide (StandGuard; Elanco Animal Health). Administration of all products was done in accordance with label instructions and Beef Quality Assurance guidelines. During processing, 13 intact bulls were discovered. Bulls were castrated by banding and monitored daily following castration. Calves (*n* = 13) that arrived as bulls were evenly distributed between treatments (*n* = 3 or 4 animals that arrived as bulls per treatment). Steers were housed in forty-eight 4.57 × 13.24 m partially covered dirt feedlot pens. Cattle housed in 2 adjacent pens shared a 76-L concrete water fountain (model J 360-F; Johnson Concrete, Hastings, NE). Cattle were revaccinated for viral and bacterial respiratory pathogens on d 0 (Nuplura PH and Titanium 5 + PH-M; Elanco Animal Health). Cattle were reimplanted on d 28 (Component TE-S; Elanco Animal Health).

Steers were monitored daily for health status as described by Wilson et al. (2016) and were treated according to standard WSBRC protocol, if necessary. No cattle were treated for suspected bovine respiratory disease (BRD). Several cattle were treated for lameness associated with foot rot or other hoof issues over the last 60 d of the trial. Further investigation did not reveal any correlation to dietary TM treatment with a similar percentage of steers from all experimental treatments being treated. A total of 8 cattle died or were removed from the experiment for reasons also believed to be unrelated to treatment. One steer, on the CON treatment, expired on d-5 from apparent bloat. On d 28, prior to data collection, a steer on the 4X treatment was discovered with its head stuck in a fence line gate. The gate was cut to remove the steer, but too much time had passed, and neurological damage had occurred. The steer was humanely euthanized 2 d later. One steer from the CON treatment and 2 steers from the 1X treatment, were removed from the experiment due to apparent neurological issues. Two cattle from the 2X treatment, were discovered dead in the pen with no further cause revealed after necropsy. One steer on the CON treatment was discovered dead in the pen and an enlarged heart observed during necropsy.

### Diets and Feed Management

A total of 48 pens were used for this experiment, with 12 pens per treatment and 5 cattle per pen (*n* = 240 animals). Within block, cattle were randomly assigned to each pen and experimental TM treatments were randomly assigned to pens within each block. 

All steers were fed a common receiving diet for 13 to 15 d (G1 = 13 d; G2 = 15 d) to allow cattle to acclimate to the feedlot environment (Table 1). After being fed a receiving diet for 13 to 15 d, cattle were gradually transitioned to a high grain finishing diet through a series of 4 step-up diets during a 28-d transitioning period. Diets were transitioned every 7 d, prior to experimental treatment administration (Table 1) on d 0. During the 13 to 15 d when the cattle were fed a receiving diet, cattle received no vitamin or mineral supplement of any kind.

**Table 1.** Ingredient and nutrient composition of receiving and transition diets

Ingredient, % of DM	Diet^*a*^				
	RCV	Step 1	Step 2	Step 3	Step 4
Rolled corn	16.7	25.9	36.6	46.9	57.7
Prairie hay	30.2	24.4	20.7	16.9	12.9
Sweet Bran^*b*^	53.1	44.7	37.7	31.2	24.4
Pelleted supplement^*c*^	―	5.0	5.0	5.0	5.0

Nutrient composition, DM basis^*d*^	RCV	Step 1	Step 2	Step 3	Step 4
Dry matter, %	67.8	71.0	73.5	74.4	77.7
Crude protein, %	19.2	15.8	14.4	14.0	12.9
Acid detergent fiber, %	23.0	19.9	19.8	20.3	15.8
TDN%^*e*^	69.8	73.0	73.1	72.6	77.3
NE_m_^*f*^ Mcal/kg	1.63	1.72	1.74	1.72	1.87
NE_g_^*g*^ Mcal/kg	1.01	1.10	1.12	1.10	1.23
Ca%	0.20	0.86	0.68	0.59	0.70
P%	0.91	0.66	0.68	0.55	0.48
Mg%	0.39	0.29	0.27	0.27	0.21
K%	1.31	1.30	1.23	1.20	0.98

^*a*^RCV = common receiving diet fed to all cattle for 13 or 15 d, Step 1 = diet fed from d-28 through d-22 for all cattle, Step 2 = diet fed from d-21 through d-15 for all cattle, Step 3 = diet fed from d-14 through d-8 for all cattle, Step 4 = diet fed from d-7 through d-1 for all cattle.

^*b*^Cargill Inc., Dalhart, TX.

^*c*^Pelleted supplement – CON supplement contained no added trace minerals but contained ground corn, wheat midds, 0.65% Ca, 0.30% NaCl, 4,715 IU/kg of Vitamin A, 25.1 IU/kg of Vitamin E, 142 IU/kg of Vitamin D, 30 g/ton of monensin (Rumensin 90; Elanco Animal Health, Greenfield, IN), and 9 g/ton of tylosin (Tylan; Elanco Animal Health, Greenfield, IN).

^*d*^Diets analyzed by Servi-Tech Laboratories: Dodge City, KS.

^*e*^Total digestible nutrients.

^*f*^Net energy maintenance.

^*g*^Net energy gain.

During the 28-d transitioning period, all cattle received only the negative control (CON) supplement (no added TM) that was formulated to meet macro mineral deficiencies present from the basal finishing diet. As a result, cattle on all experimental treatments received no TM supplementation from the time of arrival until d 0 when experimental TM treatments were initiated. This resulted in a period of depletion where cattle were not receiving any TM supplementation for at least 42 d (arrival, 13 to 15 d of receiving, and 28 d of transition) prior to experimental treatment administration.

All treatment supplements contained 0.65% Ca, 0.30% NaCl, 4,715 IU/kg of Vitamin A, 25.1 IU/kg of Vitamin E, 142 IU/kg of Vitamin D, 30 g/ton of monensin (Rumensin 90, Elanco Animal Health), and 9 g/ton of tylosin (Tylan, Elanco Animal Health). On a DM basis, finishing diets consisted of 7% hay, 15% Sweet Bran, 65% rolled corn, 5% liquid supplement, 1% urea, and 5% pelleted vitamin and treatment supplement (Table 2). Twenty-eight days prior to harvest, ractopamine hydrochloride (Optaflexx 45; Elanco Animal Health) was included in the diet (calculated average ractopamine hydrochloride intake = 390 mg·steer^−1^·d^−1^).

**Table 2.** Nutrient composition of treatment finishing diets

Ingredient composition, % DM basis	Treatment^*a*^			
	CON	1X	2X	4X
Rolled corn	65.0	65.0	65.0	65.0
Prairie hay	7.0	7.0	7.0	7.0
Sweet Bran^*b*^	15.0	15.0	15.0	15.0
Liquid supplement^*c*^	5.0	5.0	5.0	5.0
Urea	1.0	1.0	1.0	1.0
Trace mineral supplement^*d*^	5.0	5.0	5.0	5.0

Nutrient composition^*e*^, % DM basis	CON	1X	2X	4X
Dry matter, %	79.6	79.8	79.4	79.9
Crude protein, %	14.0	14.7	14.6	14.4
Acid detergent fiber, %	10.5	11.3	10.8	10.4
TDN%^*f*^	87.7	86.4	87.6	88.0
NE_m_^*g*^ Mcal/lb	2.17	2.13	2.17	2.18
NE_g_^*h*^ Mcal/lb	1.49	1.46	1.49	1.50
Ca %	0.59	0.61	0.64	0.57
P %	0.42	0.46	0.45	0.44
Mg %	0.19	0.21	0.20	0.20
K%	0.88	0.93	0.91	0.89

^*a*^Treatments included a control (**CON**; no supplemental trace minerals), **1X** (supplement containing approximately 0.15 mg/kg of Co, 10 mg/kg of Cu, 50 mg/kg of Fe, 0.5 mg/kg of I, 20 mg/kg of Mn, 0.1 mg/kg of Se, and 30 mg/kg of Zn, on a DM basis), **2X** (supplement containing approximately 0.30 mg/kg of Co, 20 mg/kg of Cu, 50 mg/kg of Fe, 1.0 mg/kg of I, 40 mg/kg of 1Mn, 0.2 mg/kg of Se, and 60 mg/kg of Zn, on a DM basis), or **4X** (supplement containing approximately 0.60 mg/kg of Co, 40 mg/kg of Cu, 50 mg/kg of Fe, 2.0 mg/kg of I, 80 mg/kg of Mn, 0.3 mg/kg of Se, and 120 mg/kg of Zn, on a DM basis).

^*b*^Cargill Inc., Dalhart, TX.

^*c*^Liquid supplement was formulated to contain (% DM basis) 45.86% corn steep, 36.17% cane molasses, 6.00% hydrolyzed vegetable oil, 5.46% 80/20 vegetable oil blend, 5.20% water, 1.23% urea (55% solution), and 0.10% xanthan gum.

^*d*^Trace mineral supplements contained ground corn, wheat midds, 0.65% Ca, 0.30% NaCl, 4,715 IU/kg of Vitamin A, 25.1 IU/kg of Vitamin E, 142 IU/kg of Vitamin D, 30 g/ton of monensin (Rumensin 90; Elanco Animal Health, Greenfield, IN), and 9 g/ton of tylosin (Tylan; Elanco Animal Health, Greenfield, IN) and the trace mineral treatments described above.

^*e*^Diets analyzed by Servi-Tech Laboratories: Dodge City, KS.

^*f*^ Total digestible nutrients.

^*g*^Net energy maintenance.

^*h*^Net energy gain.

Feeding procedures were similar to those described by Warner et al. (2020). At 0500 h each morning, feed bunks were visually evaluated to determine the amount of feed remaining from the previous day. The amount of feed to be delivered that day was adjusted based on this evaluation. Cattle were fed once daily at 1,000 h. Feed was mixed and delivered using a trailer-mounted feed mixer (274-12B feed mixer; Roto-mix, Dodge City, KS). Experimental treatment supplements and urea were weighed by hand separately from other ingredients, individually added to the mixer, and mixed into the complete ration. Treatment supplements were bagged and color coded to ensure proper addition of experimental treatments. Mixers were cleaned of all remaining ration prior to mixing the CON ration and to mixing subsequent experiment rations. Treatment supplements were weighed in individual containers and added to the mixer after all other feedstuffs. Feeding and mixing of rations were completed in order of increasing supplementation levels to decrease contamination. Pens were also individually labeled and color coded to ensure proper feeding of experimental treatments.

Diet samples were collected twice weekly, and DM was calculated after samples were dried in a forced air oven at 60 °C for 48 h. A monthly composite was created after DM was calculated and stored in a freezer until nutrient analysis could be completed. Feed refusals were weighed back before feeding on d 0, 14, 28, 56, 84, 112, 140, and 168 or if excessive orts remained in the bunk, refusal samples were dried to determine DM content and were subtracted from DM delivered in order to calculate dry matter intake (DMI).

### Experimental Treatment Trace Mineral Supplements

Dietary TM treatments were modeled from the NASEM (2016) requirements and recommendations from the 2015 consulting feedlot nutritionist survey (Samuelson et al., 2016). The survey reported mean levels of TM as follows: 0.82 mg Co/kg, 17.0 mg/kg of Cu, 13.8 mg Fe/kg, 0.73 mg I/kg, 47.9 mg Mn/kg, 0.24 mg Se/kg, and 87.3 mg Zn/kg (dry matter; DM basis). Treatment TM supplements included a negative control (**CON**) in which cattle received no supplemental TM. This was the same supplement feed to all cattle during the depletion period, a treatment designed to meet NASEM (2016) TM requirements (**1X**; 15.0 mg Co/kg, 10.0 mg Cu/kg, 50.0 mg Fe/kg, 0.50 mg I/kg, 20.0 mg Mn/kg, 0.10 mg Se/kg, and 30.0 mg Zn/kg; DM basis), a 2 times NASEM (2016) TM requirements treatment (**2X**), and 4 times NASEM (2016) TM requirements (**4X**) treatment. Exceptions to the 1X, 2X, and 4X experimental treatment structure were Fe and Se. Iron was kept constant at 50 mg/kg (DM basis) for 1X, 2X, and 4X treatments based on consulting feedlot nutritionist survey recommendations (Samuelson et al., 2016). Selenium was included at 0.1, 0.2, and 0.3 mg/kg (DM basis) for 1X, 2X, and 4X treatments, respectively due to federal Se regulations. Pelleted TM supplements were formulated with ground corn and wheat midds. Mineral sources included cobalt carbonate (CoCO_3_), copper sulfate (CuSO_4_), ferrous sulfate (FeSO_4_), ethylenediamine dihydriodide (EDDI), manganese oxide (MnO), sodium selenite (Na_2_SeO_3_), and zinc sulfate (ZnSO_4_).

### Data Collection

Individual BW were recorded for all steers on d 0,14, 28, 56, 84, 112, and prior to shipping for harvest. Body weights were measured before feeding at approximately 0400 h with no withdrawal from feed or water. All BW were adjusted using a calculated 4% shrink (BW × 0.96). Average daily gain (ADG) was calculated by dividing shrunk individual BW gain in kg by days on feed (DOF) for each period. Pen ADG was calculated as the average of individual ADG for each steer in the pen for that period. Dry matter intake was calculated from total DM feed delivery for the pen for that period divided by days on feed (DOF) and number of steers in the pen for each period (head days). Gain to feed (G:F) ratio was calculated by dividing pen ADG by pen average daily DMI for each period.

The data from the 8 steers that died or were removed from the experiment were excluded from all analyses (deads out data). Since feed intake was measured on a pen basis not on an individual animal basis, feed intake data were corrected by removing the average daily DMI for each steer removed from the pen until the respective steer ceased gaining BW. From the time the steer ceased gaining BW until the steer was physically removed from the pen and simultaneously the experiment, maintenance DMI were estimated and removed using the diet NE_m_ concentration and the NASEM (2016) equation where net energy maintenance (NE_m_) = 0.077 (shrunk BW)^0.75^.

### Serum and Liver Sampling

A subset of steers (48 animals; 1 animal/pen; 12 animals/treatment) was used to obtain blood and liver biopsy samples prior and subsequent to administration of TM supplementation treatments to examine the impact of the TM treatments on TM status and antibody concentrations. All blood samples were collected via the jugular venipuncture and stored on ice prior to harvesting serum. On d 0, 14, 28, 56, and 7 d prior to shipping to harvest, two 6-mL blood samples were collected into a tube containing silicone (BD Vacutainer; Franklin Lakes NJ) for TM analysis. On d 0, 14, 28, and 56, two 6 mL blood samples were collected into a tube containing a positive gel barrier and silicone glass powder (Medtronic; Fridley, MN) for antibody concentration analysis. Blood was allowed to clot for an average of 2 h before centrifuging. All blood tubes were centrifuged at 1,294 × g for 30 min at 4 °C (Sorvall RC6; Thermo Scientific, Waltham, MA). Serum collected for TM analysis was collected and shipped overnight to the Texas A & M Veterinary Medical Diagnostic Laboratory (TVMDL) for further analysis. Serum collected for antibody concentration analysis was stored in a −80 °C freezer until analysis. Samples were analyzed for bovine viral diarrhea virus (BVDV) types 1A, 1B, and 2, bovine herpesvirus type 1 (BHV-1), and bovine parainfluenza 3 (PI-3) virus. All serum samples for antibody concentration analysis were sent to TVMDL for analysis after the completion of the experiment.

On d 0, 28, 56, and 7 d prior to harvest, a 100-mg liver sample was collected using the procedure described by Sexten et al. (2012) and Wilson et al. (2016) with slight modifications. To obtain the biopsies, steers were briefly restrained in a hydraulic squeeze chute, hair was removed from the biopsy site (area between the 11th and 12th ribs on the right side of the animal) using surgical clipper blades, and a local anesthetic (lidocaine HCl, 20 mg/mL, 5 mL/biopsy site) was administered after a preliminary scrub with iodine. Lidocaine was injected first intramuscularly and then subcutaneously.

The biopsy site was surgically scrubbed 3 times with a commercially-available iodine scrub (Betadine; Avrio Health L.P., Stamford, CT) followed by rinsing with a 70% isopropyl alcohol solution. After the third scrub and rinse, a commercial iodine solution was sprayed on the injection sites prior to obtaining a biopsy. After ensuring the biopsy sites were thoroughly anesthetized (an additional 5 mL of lidocaine HCl was used in some instances if the biopsy site was not thoroughly anesthetized), a scalpel was utilized to make an approximately 1-cm stab incision for the insertion of the biopsy needle. The incision was made between the 11th and 12th ribs approximately 25 cm lateral to the vertebrae, and a 2.1 mm × 15.2 cm 14-gauge Tru-cut biopsy needle was inserted and then directed cranially and ventrally toward the opposite elbow. The biopsy needle was advanced through the peritoneum, the diaphragm, and then into the liver to obtain an approximately 100-mg sample of liver tissue. The incision site was closed with veterinary glue to prevent infection and sprayed again with iodine. Animals were observed twice daily for 7 d after the procedure. The 100-mg liver sample was transferred to a 2 mL centrifuge tube and stored on ice. Liver samples were shipped overnight to TVMDL for TM analysis.

### Harvest and Carcass Evaluation

Cattle were shipped approximately 116 km to a commercial abattoir in Arkansas City, Kansas in 3 harvest groups. The 5 heaviest blocks were shipped on d 126 of the experiment, the 4 middle blocks were shipped on d 140, and the 3 lightest blocks were shipped on d 156. Carcass data were collected by abattoir trained personnel and via carcass camera imaging because of COVID-19 protocols.

### Feed Analysis

For all rations, a single 400-g sample from the middle of the feed batch was collected from the mixer twice weekly. A 400-g sample of the dietary TM supplements were collected twice weekly. Within each month, the twice weekly finishing diet and TM supplement samples were individually composited and stored until analysis. Monthly composite samples of finishing diets and TM supplements, as well as composites of receiving and transition diets, were ground through a 2mm screen (Pulverisette 19, Fritsch; Pittsboro, NC), and were composited for each diet and TM supplement. Approximately 200 g of each diet and supplement composite were sent in duplicate to a commercial laboratory for proximate and TM analysis (Table 2.2; Servi-Tech, Dodge City, KS).

### Trace Mineral Tissue and Serum Analysis

Liver tissue samples and serum samples were analyzed for Co, Cu, Fe, Mn, Mo, Se, and Zn concentrations using inductively coupled plasma mass spectrometry (ICP-MS) at TVMDL (College Station, TX). In brief, samples were weighed and combined with 5 mL HNO_3_, 0.25 mL HCl, and a glass encased stir bar. One mL of H_2_O_2_ was added. The sample was then stirred for approximately 10 min and transferred to an automated microwave digester (Discover SP-D 80; CEM Corporation, Matthews, NC). The sample was then transferred to a tube and brought to 50 mL with tissue sample diluent (2% HNO_3_ and 1 µg/mL ISTD), inverted, and assayed. Aliquots of serum were diluted 1:10 with a serum diluent (2% methanol, 0.5% HNO_3_, 0.1% HCl, 0.05% Triton X-100, 50 ng/mL ISTD). Samples were then vortexed and assayed. All samples were analyzed on a PerkinElmer NexION 350x ICP-MS using Syngistix 1.1 software.

### Antibody Concentration Analysis

Serum samples were analyzed for antibody concentrations specific to BRD, specifically BVDV types 1A, 1B, and 2, infectious bovine rhinotracheitis (IBR) specifically bovine herpes virus 1 (BVH-1) titers, and parainfluenza 3 (PI-3) using the serum neutralization test in the virology section at the TVMDL (Canyon, TX). In brief, a 2-fold serial dilution was performed for all serum. Diluted samples were challenged with aliquots of specific stock virus. Samples are agitated and incubated at 37 ± 1 °C, in a humidified 5 ± 1% CO_2_ atmosphere. After 72 to 96 h of incubation, depending on virus; samples were analyzed. A serum dilution was considered positive if there is a 100% reduction in the amount of virus in the serum test wells compared to that present in the positive control wells. The highest dilution of serum resulting in complete neutralization of virus is the end point titer for that serum. All titers are reported as the final serum dilution prior to the addition of the cell culture suspension. Titers are reported as the reciprocal of the dilution.

### Statistical Analysis

This experiment was organized in a randomized complete block design and blocked by BW within group. For all performance measurements, pen served as the experiential unit (*n* = 48). All performance and carcass characteristic data were analyzed using the MIXED procedure of SAS 9.4 (SAS Institute Inc.; Cary, NC) with treatment as a fixed effect and block as a random effect. There was no effect of treatment in the overall model for any performance or carcass parameters (*P* ≥ 0.12), so predetermined contrasts were evaluated including CON vs. TM supplement (SUPP) and the linear and quadratic effects of TM supplementation. Due to unequal spacing in dietary treatments, contrast coefficients were determined using the IML procedure of SAS.

Trace mineral serum and liver concentrations and antibody concentration data were analyzed using the GLIMMIX procedure of SAS as a repeated measure with treatment, day, and treatment × day as fixed effects and block as a random effect. Covariance structures within the model were compared. The appropriate covariance structure was chosen based off the lowest Akaike Information Criterion (AIC). Analysis for Co liver, Cu serum and liver, Se serum concentrations and BVDV Type 1B, 2, and BHV-1 antibody titers utilized an unstructured covariance structure. Analysis for Co serum, Fe liver, Mn serum, and Se liver concentrations utilized a heterogeneous autoregressive (1) covariance structure. Day was included as a repeated measure with pen as the subject. All data from steers removed from the experiment were excluded from statistical analysis. Significance was determined when *P* ≤ 0.05 and tendencies were considered when *P* > 0.05 and *P* ≤ 0.10.

## RESULTS AND DISCUSSION

### Trace Mineral Inclusion from the Experimental Trace Mineral Supplements

Experimental diets were formulated to be identical except for the TM composition of the treatment supplements. Proximate analysis of diets excluding TM is presented in Table 2. The consulting feedlot nutritionist survey reported that most feedlot nutritionists do not take TM from the basal diet into consideration when formulating (Samuelson et al., 2016). Therefore, the objective of this experiment was to create supplements that reflected nutritional practices within the commercial feedlot industry. Based off the actual supplement inclusion rate (5% of diet DM) and the TM analysis of the treatment supplements (Table 3), the actual TM inclusion level in the final diets was calculated (Table 4). The 1X supplement resulted in a supplementation of 0.09 mg/kg of Co, 7.60 mg/kg of Cu, 42.6 mg/kg of Fe, 14.0 mg/kg of Mn, 0.09 mg/kg of Se, and 23.7 mg/kg of Zn on a DM basis when compared to the basal (CON) diet. The 2X supplement resulted in a supplementation of 0.13 mg/kg of Co, 18.6 mg/kg of Cu, 64.9 mg/kg of Fe, 34.9 mg/kg of Mn, 0.20 mg/kg of Se, and 66.8 mg/kg of Zn on a DM basis when compared to the basal (CON) diet. The 4X supplement resulted in a supplementation of 0.49 mg/kg of Co, 36.2 mg/kg of Cu, 68.1 mg/kg of Fe, 70.9 mg/kg of Mn, 0.26 mg/kg of Se, and 107.5 mg/kg of Zn on a DM basis when compared to the basal (CON) diet. Trace mineral analysis of the experimental treatment supplements and the supplement inclusion rate yielded TM supplementation levels within the targeted range of the experimental design. Total dietary TM inclusion from the basal diet and the supplement inclusion is presented in Table 4. While consulting nutritionists commonly do not take TM from the basal diet into consideration, it should be noted, the CON diet (no TM supplementation) still provided sufficient levels of almost all TM, with the exception of Cu, to meet NASEM (2016) TM requirements. Copper levels (6.5 mg/kg) in the CON diet were below the NASEM (2016) requirements (10 mg/kg).

**Table 3.** Analyzed mineral composition of treatment supplements

Mineral composition^*b*^, DM	Treatment^*a*^			
	CON	1X	2X	4X
Calcium, %	14.1	14.0	14.5	15.4
Phosphorus, %	0.44	0.45	0.41	0.38
Magnesium, %	0.28	0.28	0.26	0.28
Potassium, %	0.52	0.53	0.49	0.48
Sulfur, %	0.16	0.23	0.32	0.38
Sodium, %	2.50	2.64	2.90	2.70
Cobalt, mg/kg	1.10	2.90	3.60	10.9
Copper, mg/kg	32.5	184.5	404	756
Iron, mg/kg	653	1,505	1,950	2,015
Manganese, mg/kg	183	463	880	1,600
Selenium mg/kg	5.10	6.85	9.00	10.3
Zinc, mg/kg	100	573	1,435	2,250

^*a*^Treatments included a control (**CON**; no supplemental trace minerals), **1X** (supplement containing approximately 0.15 mg/kg of Co, 10 mg/kg of Cu, 50 mg/kg of Fe, 0.5 mg/kg of I, 20 mg/kg of Mn, 0.1 mg/kg of Se, and 30 mg/kg of Zn, on a DM basis), **2X** (supplement containing approximately 0.30 mg/kg of Co, 20 mg/kg of Cu, 50 mg/kg of Fe, 1.0 mg/kg of I, 40 mg/kg of Mn, 0.2 mg/kg of Se, and 60 mg/kg of Zn, on a DM basis), or **4X** (supplement containing approximately 0.60 mg/kg of Co, 40 mg/kg of Cu, 50 mg/kg of Fe, 2.0 mg/kg of I, 80 mg/kg of Mn, 0.3 mg/kg of Se, and 120 mg/kg of Zn, on a DM basis).

^*b*^Diets analyzed by Servi-Tech Laboratories: Dodge City, KS.

**Table 4.** Total dietary trace mineral inclusion based on analyzed treatment supplements

Mineral composition^*b*^, DM	Treatment^*a*^			
	CON^*c*^	1X	2X	4X
Cobalt mg/kg	0.15	0.24	0.28	0.64
Copper mg/kg	6.50	14.1	25.1	42.7
Iron mg/kg	119	161	183	187
Manganese mg/kg	30.5	44.5	65.4	101
Selenium mg/kg	0.25	0.34	0.45	0.51
Zinc mg/kg	44.5	68.2	111	152

^*a*^Treatments included a control (**CON**; no supplemental trace minerals), **1X** (supplement containing approximately 0.15 mg/kg of Co, 10 mg/kg of Cu, 50 mg/kg of Fe, 0.5 mg/kg of I, 20 mg/kg of Mn, 0.1 mg/kg of Se, and 30 mg/kg of Zn, on a DM basis), **2X** (supplement containing approximately 0.30 mg/kg of Co, 20 mg/kg of Cu, 50 mg/kg of Fe, 1.0 mg/kg of I, 40 mg/kg of Mn, 0.2 mg/kg of Se, and 60 mg/kg of Zn, on a DM basis), or **4X** (supplement containing approximately 0.60 mg/kg of Co, 40 mg/kg of Cu, 50 mg/kg of Fe, 2.0 mg/kg of I, 80 mg/kg of Mn, 0.3 mg/kg of Se, and 120 mg/kg of Zn, on a DM basis).

^*b*^Total trace mineral inclusion in the treatment diets was calculated from the analyzed CON diet trace mineral composition and analyzed treatment supplements 1X, 2X, and 4X (Table 3).

^*c*^CON diet was analyzed for trace mineral content by Servi-Tech Laboratories: Dodge City, KS.

### Performance

The effects of supplemental TM on steer performance and DMI are presented in Table 5. There was no difference in BW (CON vs. SUPP *P* ≥ 0.18) or ADG (CON vs. SUPP *P* ≥ 0.11) due to TM supplementation. Furthermore, there was no linear or quadratic trend in BW (Linear *P* ≥ 0.17; Quadratic *P* ≥ 0.17) or ADG among treatments (Linear *P* ≥ 0.12; Quadratic *P* ≥ 0.38). There was no difference in DMI during from d 0 to d 13 of the experiment due to TM supplementation (CON vs. SUPP *P* = 0.39). Interestingly, from d 14 to d 27, DMI for cattle consuming the CON treatment was less than (*P* = 0.04) cattle fed 1X, 2X, and 4X. Over the next several intervals, cattle fed the CON diet had average DMI that did not differ from that of the other 3 TM treatments (*P* ≥ 0.58). During the last 28 d of the experiment, when a beta agonist was included in the diet, cattle consuming the CON diet tended (*P* = 0.08) to have decreased DMI than cattle consuming the 1X, 2X, or 4X diets. There was no difference in overall DMI between cattle consuming the CON diet and cattle consuming the 1X, 2X, or 4X diets (*P* = 0.99). While TM supplemented cattle were also more efficient during the final 28 d, when a beta agonist was included (*P* = 0.03), compared with CON cattle, there was no difference in G:F for any other interval (*P* ≥ 0.31) or overall (*P* = 0.48) for cattle offered the CON diet compared with cattle offered the 1X, 2X, or 4X diets.

**Table 5.** Effects of increasing levels of trace mineral supplementation on performance in feedlot steers

Item	Treatment^*a*^					*P*-value		
	CON	1X	2X	4X	SEM^*b*^	Linear	Quad	CON vs. SUPP
BW^*c*^, kg								
Initial^*d*^	288	288	289	288	7.9	0.85	0.85	0.71
d 0	371	369	367	367	8.3	0.53	0.53	0.21
d 14	394	391	395	392	8.2	0.98	0.98	0.50
d 28	432	432	433	433	8.7	0.91	0.91	0.82
d 56	499	499	495	496	8.9	0.56	0.56	0.53
d 84	548	543	542	543	8.7	0.32	0.32	0.21
β-agonist^*e*^	574	568	567	570	7.5	0.17	0.17	0.18
Final^*d*^	592	592	590	596	7.4	0.58	0.58	0.90
ADG^*f*^, kg								
d 0 – 13	1.67	1.54	1.98	1.81	0.189	0.23	0.44	0.50
d 14 – 27	2.71	2.93	2.77	2.95	0.149	0.31	0.91	0.25
d 28 – 55	2.37	2.40	2.18	2.26	0.070	0.12	0.34	0.25
d 56 – 83	1.76	1.59	1.70	1.68	0.075	0.72	0.38	0.20
d 84 – β-agonist	1.03	1.04	1.09	1.12	0.113	0.36	0.93	0.53
β-agonist – final	0.65	0.87	0.84	0.93	0.168	0.16	0.49	0.11
d 0 – final	1.61	1.62	1.63	1.67	0.045	0.20	0.75	0.47
DMI^*h*^, kg/d								
d 0 – 13	10.7	10.8	11.1	10.8	0.31	0.68	0.23	0.39
d 14 – 27	12.3	12.8	12.8	12.8	0.30	0.17	0.11	0.04
d 28 – 55	13.7	14.1	13.7	13.6	0.24	0.40	0.39	0.58
d 56 – 84	13.2	13.1	13.3	13.0	0.25	0.68	0.65	0.79
d 84 – β-agonist	12.2	11.9	12.1	12.3	0.38	0.49	0.28	0.65
β-agonist – final	10.1	10.6	10.6	10.7	0.33	0.13	0.34	0.08
d 0 – final	12.2	12.1	12.2	12.3	0.24	0.51	0.55	0.99
G:F^*i*^								
d 0 – 13	0.133	0.138	0.162	0.156	0.0196	0.16	0.52	0.32
d 14 – 27	0.206	0.248	0.197	0.236	0.0174	0.48	0.79	0.31
d 28 – 55	0.164	0.166	0.163	0.164	0.0079	0.94	0.95	0.94
d 56 – 84	0.134	0.120	0.133	0.128	0.0098	0.92	0.78	0.56
d 84 – β-agonist	0.077	0.087	0.084	0.090	0.0110	0.39	0.82	0.43
β-agonist – final	0.055	0.087	0.086	0.085	0.0120	0.08	0.13	0.03
d 0 – final	0.132	0.134	0.134	0.136	0.0031	0.34	0.98	0.48

^*a*^Treatments included a control (**CON**; no supplemental trace minerals), **1X** (supplement containing 0.15 mg/kg of Co, 10 mg/kg of Cu, 50 mg/kg of Fe, 0.5 mg/kg of I, 20 mg/kg of Mn, 0.1 mg/kg of Se, and 30 mg/kg of Zn), **2X** (supplement containing 0.30 mg/kg of Co, 20 mg/kg of Cu, 50 mg/kg of Fe, 1.0 mg/kg of I, 40 mg/kg of Mn, 0.2 mg/kg of Se, and 60 mg/kg of Zn), or **4X** (supplement containing 0.60 mg/kg of Co, 40 mg/kg of Cu, 50 mg/kg of Fe, 2.0 mg/kg of I, 80 mg/kg of Mn, 0.3 mg/kg of Se, and 120 mg/kg of Zn).

^*b*^ *n* = 48 pens; pens per treatment = 12.

^*c*^Body weight adjusted by a 4% calculated pencil shrink.

^*d*^Initial BW is an average of arrival BW and BW at processing.

^*e*^Cattle were administered a β-agonist (Optaflexx; Elanco Animal Health, Greenfield, IN) 28 d prior to harvest.

^*f*^Cattle were harvested in 3 groups; d 126 (*n* = 20 pens; 5 pens per treatment), d 140 (*n* = 16 pens; 4 pens per treatment), and d 154 (*n* = 12 pens; 3 pens per treatment).

^*g*^Pen average daily gain calculated from the average of individual shrunk BW gain divided by days on feed in each period.

^*h*^Pen dry matter intake calculated from total DMI for the pen for each period divided by the total steers and days on feed in each period.

^*i*^Gain: feed calculated by dividing the ADG for the pen by the average daily DMI for the pen for each respective period.

Previous research involving individual TM supplementation has been variable (Engle and Spears, 2000, 2001; Legleiter et al., 2005; Malcolm-Callis et al., 2000; Perry et al., 1976; Tiffany et al., 2003). Variation in experimental designs and objectives of TM studies differ as well. There have been limited published experiments with similar dietary treatments to the current experiment; however, these experiments involved other factors such as TM source and implant status. Berrett et al. (2015) evaluated no supplemental TM, TM offered at NRC (1996) requirements from inorganic sources, or TM offered at similar concentrations to consulting nutritionist average recommendations (Samuleson et al., 2016) from organic and inorganic sources. Final BW, ADG, DMI, and G:F were not different across treatment, regardless of supplementation, concentration, or source (Berrett et al., 2015).

Rhoads et al. (2003) investigated TM source and concentrations within source for Co, Cu, Mn, and Zn. While there was not a negative control and fewer TM were investigated, there was no difference in BW regardless of dietary treatment or a difference between inorganic sources of TM at 1.5 or 3 times the NRC (1996) requirements. There was also no difference in ADG, DMI, or G:F regardless of source or concentration of TM (Rhoads et al., 2003).

Niedermayer et al. (2018) evaluated varying levels of TM supplementation with steer implant status. There was no implant × TM interaction for cattle during the last 68 d of the finishing phase. Data were then evaluated for the main effect of TM supplementation. There was no difference in overall BW due to dietary TM treatment. Furthermore, there was no effect of TM concentration on DMI in TM supplemented steers. However, there was an effect of supplementation on DMI. Steers fed a TM supplement had a higher DMI than nonsupplemented steers. Overall ADG tended to be affected by TM supplementation while overall G:F was not affected by supplementation (Niedermayer et al., 2018).

Previous research has been conducted regarding the synergistic effects of TM and beta agonists. Results from the current experiment indicate that the addition of TM at or above NASEM (2016) requirements when combined with supplemental ractopamine hydrochloride may affect animal performance. When comparing the CON cattle to TM supplemented cattle there was a numerical increase in ADG (*P* = 0.11), a tendency for an increase in DMI (*P* = 0.08), and an increase in G:F (*P* = 0.03) for cattle receiving TM supplementation. Messersmith et al. (2021) investigated the effects of increasing Cu concentrations with or without the addition of ractopamine hydrochloride. With regards to the addition of ractopamine hydrochloride, DMI intake tended to quadratically increase due to Cu supplementation with cattle receiving 10 mg/kg having greater DMI than the remaining Cu supplementation treatments (0 and 20 mg/kg; Messersmith et al., 2021). Furthermore, the addition of ractopamine improved ADG with increasing Cu supplementation, with cattle receiving 10 mg/kg having the greatest ADG, resulting in a tendency for a quadratic increase in G:F (Messersmith et al., 2021).

With the exception of the results observed during the beta agonist period, the present experiment is in agreement with the majority of previous research, where TM supplementation above published requirements had no effect on overall feedlot cattle performance.

### Carcass Characteristics

Carcass data are presented in Table 6. There was no difference in hot carcass weight (HCW), rib eye area (REA), fat thickness (FT), marbling score, USDA Yield Grade, or dressing percentage for cattle fed the CON treatment compared to those fed a TM supplement treatment (*P* ≥ 0.30). There were also no linear or quadratic trends for any carcass characteristics (Linear *P* ≥ 0.24; Quadratic *P* ≥ 0.23).

**Table 6.** Effects of increased levels of trace mineral supplementation on carcass characteristics in feedlot steers

Item	Treatment^*a*^				*P*-value			
	CON	1X	2X	4X	SEM^*b*^	Linear	Quad	CON vs. SUPP
Hot carcass weight	382	384	381	380	4.9	0.54	0.90	0.90
Rib eye area, cm^2^	92.0	93.7	89.2	90.9	1.52	0.24	0.52	0.64
Fat thickness^*c*^, cm	1.51	1.38	1.47	1.47	0.076	0.99	0.36	0.30
Dressing percent	63.7	63.8	63.7	63.5	0.26	0.46	0.51	0.95
Marbling score^*d*^	471	476	447	474	12.1	0.90	0.23	0.69
USDA Yield Grade	3.03	2.83	3.11	3.04	0.128	0.56	0.89	0.74

^*a*^Treatments included a control (**CON**; no supplemental trace minerals), **1X** (supplement containing 0.15 mg/kg of Co, 10 mg/kg of Cu, 50 mg/kg of Fe, 0.5 mg/kg of I, 20 mg/kg of Mn, 0.1 mg/kg of Se, and 30 mg/kg of Zn), **2X** (supplement containing 0.30 mg/kg of Co, 20 mg/kg of Cu, 50 mg/kg of Fe, 1.0 mg/kg of I, 40 mg/kg of Mn, 0.2 mg/kg of Se, and 60 mg/kg of Zn), or **4X** (supplement containing 0.60 mg/kg of Co, 40 mg/kg of Cu, 50 mg/kg of Fe, 2.0 mg/kg of I, 80 mg/kg of Mn, 0.3 mg/kg of Se, and 120 mg/kg of Zn).

^*b*^ *n* = 48 pens, pens per treatment = 12.

^*c*^Fat measurement was taken between the 12th and 13th rib.

^*d*^Small^00^ = 400; Modest^00^ = 500; Moderate^00^ = 600.

These results are similar to those published by Berrett et al. (2015) who reported no difference in HCW, dressing percentage, FT, REA, USDA Yield Grade, or marbling score regardless of TM supplementation. Rhoads et al. (2003) reported no difference between cattle fed inorganic sources at 1.5- or 3-times NRC requirements for HCW, USDA Yield Grade, FT, and marbling score. However, dressing percentage was lower for cattle fed either the organic or inorganic mineral source at 1.5 times the NRC recommendation than for cattle fed either the organic NRC recommended level or at 3 times NRC recommendations through inorganic sources (Rhoads et al., 2003). Niedermayer et al. (2018) also reported no effect of TM supplementation on dressing percentage, FT, REA, or marbling score.

### Trace Mineral Liver and Serum Concentrations

Trace mineral concentrations were observed from a subset of 48 steers (1 steer per pen/12 steers per treatment). Trace mineral concentrations are reported on a wet weight basis.

**Figure 1.**
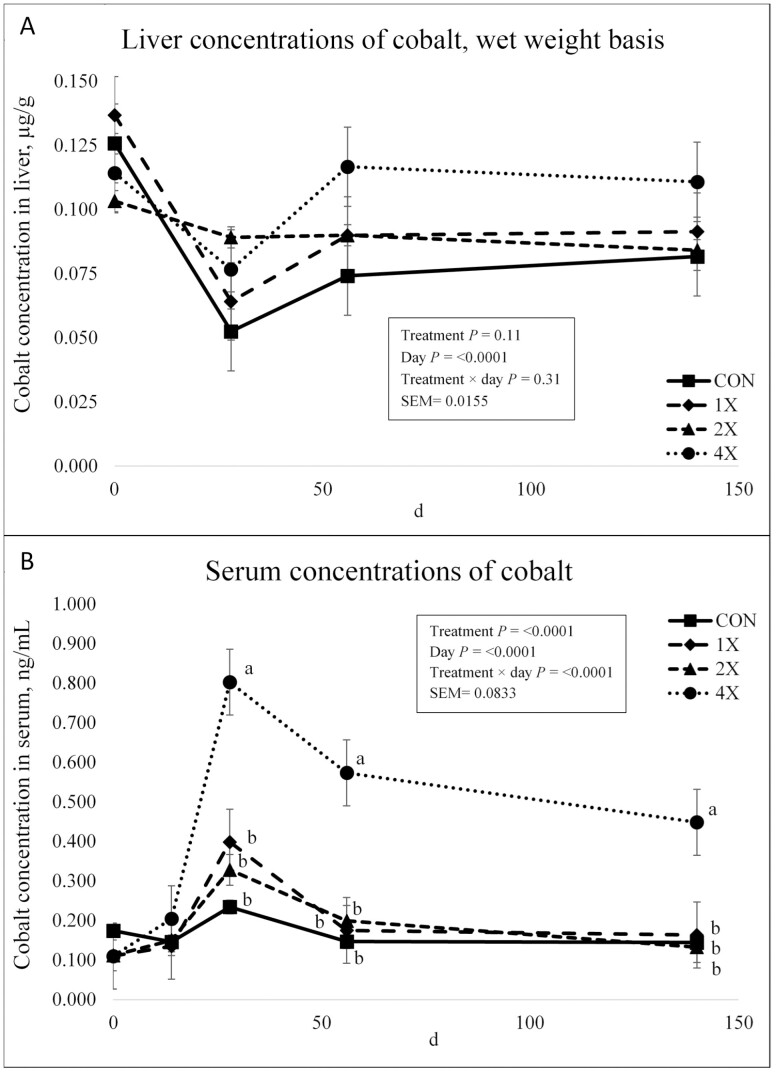
Liver (**a**) and serum (**b**) concentrations of cobalt in finishing steers consuming 1 of 4 trace mineral supplements. Treatments included a control (**CON**; no supplemental trace minerals), **1X** (supplement containing 0.15 mg/kg of Co, 10 mg/kg of Cu, 50 mg/kg of Fe, 0.5 mg/kg of I, 20 mg/kg of Mn, 0.1 mg/kg of Se, and 30 mg/kg of Zn, on a DM basis), **2X** (supplement containing 0.30 mg/kg of Co, 20 mg/kg of Cu, 50 mg/kg of Fe, 1.0 mg/kg of I, 40 mg/kg of Mn, 0.2 mg/kg of Se, and 60 mg/kg of Zn, on a DM basis), or **4X** (supplement containing 0.60 mg/kg of Co, 40 mg/kg of Cu, 50 mg/kg of Fe, 2.0 mg/kg of I, 80 mg/kg of Mn, 0.3 mg/kg of Se, and 120 mg/kg of Zn on a DM basis). *Cattle were harvested in 3 groups; d 126 (*n* =20 pens; 5 pens per treatment), d 140 (*n* = 16 pens; 4 pens per treatment), and d 154 (*n* = 12 pens; 3 pens per treatment). In this figure, 140 days on feed represents the final measurement, regardless of the actual harvest date. ^a,b,c^Means within grouping without a common superscript letter differ (*P* < 0.05). Values plotted represent least squares means ± SE of the mean, calculated for 12 animals per experimental group. Within time points, the slice output option of SAS (SAS Inst. Inc., Cary, NC) was used to perform mean separations.

Cobalt concentrations in the liver are displayed in Figure 1a. There was no treatment × day interaction (*P* = 0.31) or effect of treatment on Co concentrations (*P* = 0.11). There was an effect of day on Co concentrations in the liver (*P* < 0.0001) where concentrations for all treatments decreased on d 0 to d 28, increased on d 56, and then remained relatively constant until harvest. Kincaid et al. (2003) reported that Co supplementation in nonpregnant, nonlactating Holstein cows did not affect Co concentrations in the liver. Kincaid et al. (2003) only obtained liver samples at harvest, so no time effect was evident. In contrast to the present experiment, Niedermayer et al. (2018) reported an effect of TM supplementation on Co liver concentrations on d 70. Cattle receiving NASEM (2016) TM requirements had greater liver Co concentrations than cattle receiving the consulting nutritionist mode TM supplementation (Samuleson et al., 2016) and negative control steers. On d 124, TM supplemented cattle had greater Co liver concentrations than the control. (Niedermayer et al., 2018).

There was a treatment × day interaction for Co concentrations in serum (*P* < 0.0001) as shown in Figure 1b. There was no difference in serum Co concentration on d 0 or d 14. Serum Co concentrations for all treatments increased on d 28, decreased on d 56, and Kincaid et al. (2003) then stabilized though harvest. Cattle on the 4X treatment had greater (*P* < 0.0001) serum Co concentrations on d 28 through harvest compared to cattle on the CON, 1X, and 2X treatments, which did not differ (*P* ≥ 0.17) from d 28 through harvest. Kincaid et al. (2003) reported Co concentrations in serum of nonpregnant Holstein cows were not affected by supplemental Co. However, serum Co concentrations, regardless of Co supplementation increased with time.

**Figure 2.**
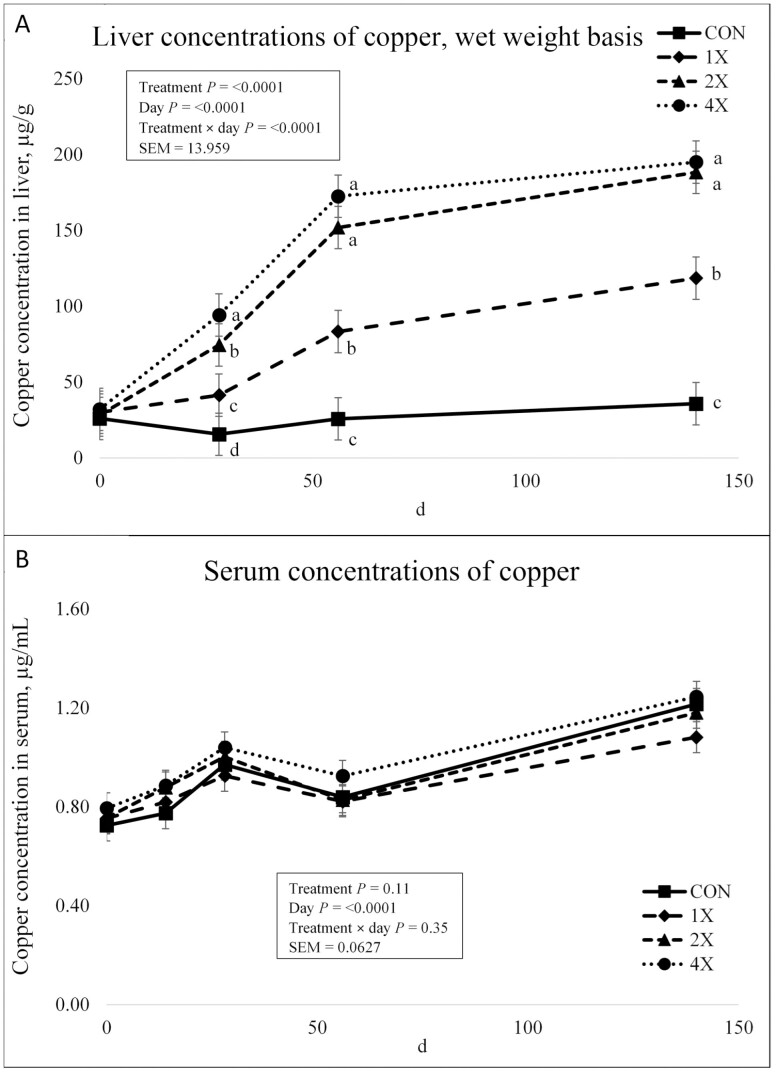
Liver (**a**) and serum (**b**) concentrations of copper in finishing steers consuming 1 of 4 trace mineral supplements. Treatments included a control (**CON**; no supplemental trace minerals), **1X** (supplement containing 0.15 mg/kg of Co, 10 mg/kg of Cu, 50 mg/kg of Fe, 0.5 mg/kg of I, 20 mg/kg of Mn, 0.1 mg/kg of Se, and 30 mg/kg of Zn, on a DM basis), **2X** (supplement containing 0.30 mg/kg of Co, 20 mg/kg of Cu, 50 mg/kg of Fe, 1.0 mg/kg of I, 40 mg/kg of Mn, 0.2 mg/kg of Se, and 60 mg/kg of Zn, on a DM basis), or **4X** (supplement containing 0.60 mg/kg of Co, 40 mg/kg of Cu, 50 mg/kg of Fe, 2.0 mg/kg of I, 80 mg/kg of Mn, 0.3 mg/kg of Se, and 120 mg/kg of Zn on a DM basis). ^*^Cattle were harvested in 3 groups; d 126 (*n* =20 pens; 5 pens per treatment), d 140 (*n* = 16 pens; 4 pens per treatment), and d 154 (*n* = 12 pens; 3 pens per treatment). In this figure, 140 days on feed represents the final measurement, regardless of the actual harvest date. ^a,b,c^Means within grouping without a common superscript letter differ (*P* < 0.05). Values plotted represent least squares means ± SE of the mean, calculated for 12 animals per experimental group. Within time points, the slice output option of SAS (SAS Inst. Inc., Cary, NC) was used to perform mean separations.

There was a treatment × day interaction (*P* < 0.0001) for Cu concentrations in the liver as shown in Figure 2a. Liver Cu concentrations did not differ on d 0 between treatments (*P* = 0.57). However, concentrations for cattle on the 1X, 2X, and 4X treatments increased steadily over the course of the experiment while cattle on the CON treatment maintained near baseline liver Cu concentrations through harvest. Liver Cu concentrations differed (*P* ≤ 0.04) between all 4 treatments on d 28 with 4X having the greatest concentrations, followed by 2X, 1X, and then the CON. Liver Cu concentrations were not different for cattle on the 4X and 2X treatments from d 56 through harvest (*P* ≥ 0.20). However, liver Cu concentrations were greater for 4X and 2X than the 1X or CON treatments and liver Cu concentrations were greater for 1X treatment than CON from d 56 through harvest (*P* ≤ 0.001). Cattle on all experimental treatments exhibited concentrations that were considered adequate (CON and 1X) or high (2X and 4X) for beef cattle (Puls, 1994). However, it should be noted that at no point during the experiment were liver Cu concentrations considered toxic for any experimental treatment (Puls, 1994). Previous research regarding concentrations of Cu from CuSO_4_ reported similar findings. Engle and Spears (2000) evaluated 0, 10, and 20 mg/kg of supplemental Cu from CuSO_4_ fed to Angus steers and reported a treatment × time interaction in liver Cu concentrations. Liver Cu concentrations were similar across treatments on d 0 and increased over time. Liver Cu concentrations were greater at harvest for steers fed 20 mg/kg of Cu than cattle consuming 10 mg/kg of Cu (Engle and Spears, 2000). Other research conducted on Simmental cattle reported a similar treatment × time interaction that indicated Cu supplementation increased liver Cu concentrations (Engle and Spears, 2001). Liver Cu concentrations of cattle supplemented with 40 mg of Cu/kg DM were greater than cattle fed 10 mg of Cu/kg DM over the finishing phase (Engle and Spears, 2001).

There was no treatment × day interaction (*P* = 0.35) or effect of treatment (*P* = 0.11) for serum Cu concentrations as shown in Figure 2b. However, there was an effect of day on serum Cu concentrations (*P* < 0.0001). Copper concentrations remained stable from d 0 to d 14. From d 14 to 28 serum Cu concentrations increased, followed by a decrease in serum concentrations from d 28 to d 56. Serum Cu concentrations increased for all treatments from d 56 through harvest. Although serum Cu concentrations fluctuated over time, cattle maintained concentrations within a narrow margin. According to Herdt and Hoff (2011), the detected values for serum Cu were within normal range for beef cattle (0.6 to 1.1 µg/mL). However, more published research evaluating Cu supplementation and blood biomarkers has reported plasma rather than serum Cu concentrations. Several studies have investigated plasma Cu concentrations resulting from various Cu supplementation levels. Published research indicates that steers supplemented with Cu had greater plasma Cu concentrations than nonsupplemented cattle at multiple sampling times (Engle and Spears 2000, 2001).

Previous research has also reported that Cu levels in serum are less than that in plasma indicating that serum Cu levels should not be directly compared to plasma Cu levels to determine Cu status (Paynter, 1982). Laven et al. (2007) compared the use of serum and plasma when determining Cu status in cattle. They determined that serum Cu concentration was not a suitable substitute for plasma Cu concentration for the detection of ‘marginal’ blood Cu status in cattle as there is variability in the amount of Cu sequestered during clotting (Laven et al., 2007). This variability and loss of Cu may cause serum to be a less effective measure of Cu status. Based on Cu liver and serum data from the present experiment, liver Cu concentrations appear to be more indicative the effects of Cu supplementation. Serum Cu concentrations remained within a tight margin during the experiment as the body has a tight homeostatic control on circulating Cu. As such, changes in serum Cu concentrations over time are more indicative of transient Cu use within the body.

**Figure 3.**
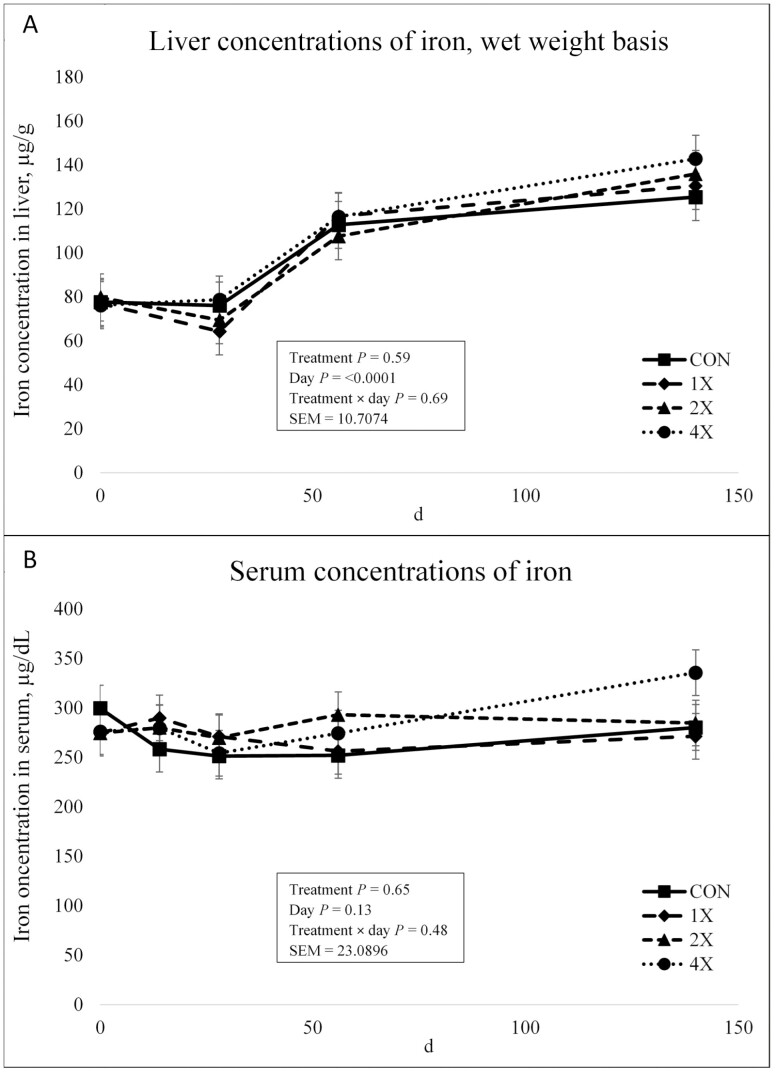
Liver (**a**) and serum (**b**) concentrations of iron in finishing steers consuming 1 of 4 trace mineral supplements. Treatments included a control (**CON**; no supplemental trace minerals), **1X** (supplement containing 0.15 mg/kg of Co, 10 mg/kg of Cu, 50 mg/kg of Fe, 0.5 mg/kg of I, 20 mg/kg of Mn, 0.1 mg/kg of Se, and 30 mg/kg of Zn, on a DM basis), **2X** (supplement containing 0.30 mg/kg of Co, 20 mg/kg of Cu, 50 mg/kg of Fe, 1.0 mg/kg of I, 40 mg/kg of Mn, 0.2 mg/kg of Se, and 60 mg/kg of Zn, on a DM basis), or **4X** (supplement containing 0.60 mg/kg of Co, 40 mg/kg of Cu, 50 mg/kg of Fe, 2.0 mg/kg of I, 80 mg/kg of Mn, 0.3 mg/kg of Se, and 120 mg/kg of Zn on a DM basis). ^*^Cattle were harvested in 3 groups; d 126 (*n* =20 pens; 5 pens per treatment), d 140 (*n* = 16 pens; 4 pens per treatment), and d 154 (*n* = 12 pens; 3 pens per treatment). In this figure, 140 days on feed represents the final measurement, regardless of the actual harvest date. Values plotted represent least squares means ± SE of the mean, calculated for 12 animals per experimental group. Within time points, the slice output option of SAS (SAS Inst. Inc., Cary, NC) was used to perform mean separations.

As expected, there was not a treatment × day interaction (*P* = 0.69) or effect of treatment (*P* = 0.59) for concentrations for Fe in the liver, as shown in Figure 3a, due to Fe being supplemented at the same rate in all TM supplement treatments. There was an effect of day on liver Fe concentrations (*P* < 0.0001). Concentrations for all for treatments were similar on d 0. On d 28, liver Fe concentrations remained stable. However, on d 56 liver Fe concentrations increased and continued to increase through harvest. Standish et al. (1969) evaluated various levels of dietary Fe on tissue mineral composition in cattle. Liver Fe reflected the level of dietary Fe. The change in liver Fe due to dietary Fe was almost totally linear for steers fed 0, 400, and 1,600 mg/kg Fe (Standish et al., 1969). If dietary levels of Fe are accurately reflected in the liver, then the response in the present experiment seems reasonable.

There was no treatment × day interaction or effect of treatment or day on Fe serum concentrations (*P* ≥ 0.13) as shown in Figure 3b. Due to no variations in supplemental Fe among supplemented treatments, this result was also expected. Koong et al. (1970) reported that an increase in dietary Fe increased serum Fe concentrations in calves. These results would support the present experiment where no differences were observed in serum Fe concentrations.

**Figure 4.**
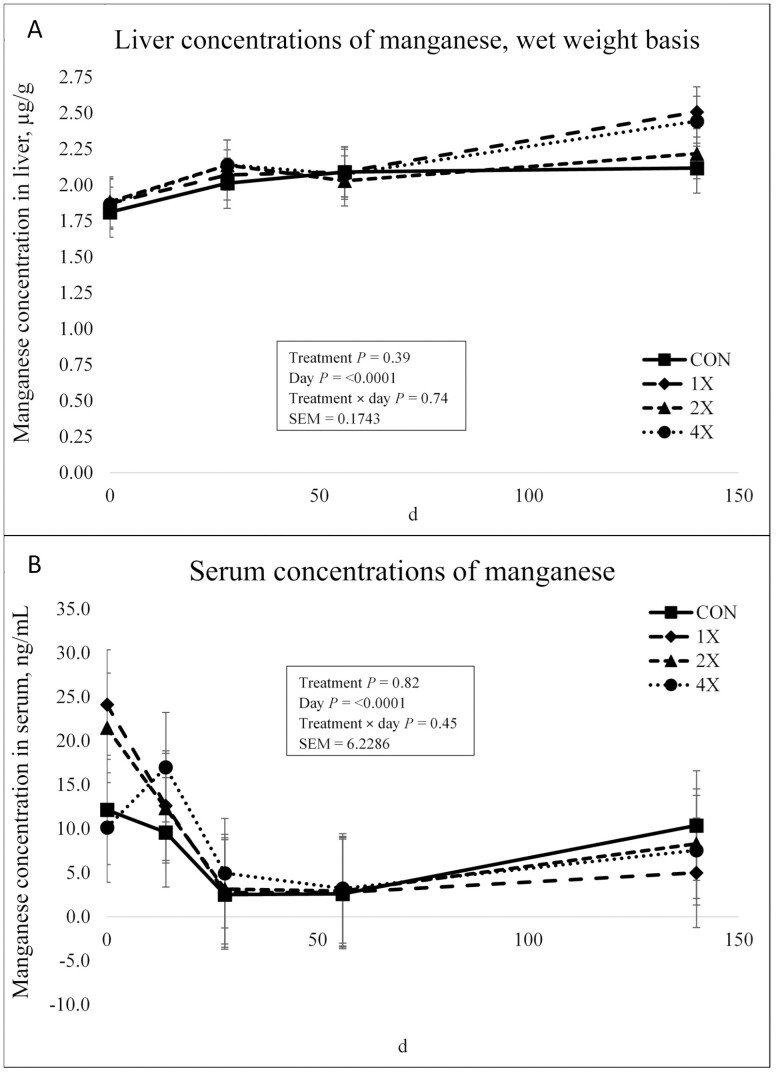
Liver (**a**) and serum (**b**) concentrations of manganese in finishing steers consuming 1 of 4 trace mineral supplements. Treatments included a control (**CON**; no supplemental trace minerals), **1X** (supplement containing 0.15 mg/kg of Co, 10 mg/kg of Cu, 50 mg/kg of Fe, 0.5 mg/kg of I, 20 mg/kg of Mn, 0.1 mg/kg of Se, and 30 mg/kg of Zn, on a DM basis), **2X** (supplement containing 0.30 mg/kg of Co, 20 mg/kg of Cu, 50 mg/kg of Fe, 1.0 mg/kg of I, 40 mg/kg of Mn, 0.2 mg/kg of Se, and 60 mg/kg of Zn, on a DM basis), or **4X** (supplement containing 0.60 mg/kg of Co, 40 mg/kg of Cu, 50 mg/kg of Fe, 2.0 mg/kg of I, 80 mg/kg of Mn, 0.3 mg/kg of Se, and 120 mg/kg of Zn on a DM basis). ^*^Cattle were harvested in 3 groups; d 126 (*n* =20 pens; 5 pens per treatment), d 140 (*n* = 16 pens; 4 pens per treatment), and d 154 (*n* = 12 pens; 3 pens per treatment). In this figure, 140 days on feed represents the final measurement, regardless of the actual harvest date. Values plotted represent least squares means ± SE of the mean, calculated for 12 animals per experimental group. Within time points, the slice output option of SAS (SAS Inst. Inc., Cary, NC) was used to perform mean separations.

There was no treatment × day interaction (*P* = 0.74) or effect of treatment (*P* = 0.39) for Mn concentrations in the liver as shown in Figure 4a. There was an effect of day on liver Mn concentrations (*P* < 0.0001). Liver Mn concentrations did not differ on d 0. On d 28, liver Mn concentrations increased, then slightly decreased on d 56. Liver Mn concentrations then increased through harvest. All liver Mn concentrations were within normal ranges indicating that Mn was neither deficient in the basal diet nor toxic in the 4X diet (Puls, 1994). In an experiment by Legleiter et al. (2005), liver samples collected directly after harvest displayed a linear increase in Mn with increasing levels of dietary Mn from MnSO_4_. Ahola et al. (2004) evaluated the effects of source of TM against a negative control in grazing beef cows over a 2-yr period. There was a treatment × year interaction for Mn concentration in the liver, where liver Mn concentrations were greater in supplemented cattle at the end of yr 1, but, lower in cattle supplemented with Mn at the end of yr 2 compared to control cattle. Liver Mn concentrations were not affected by TM source (Ahola et al., 2004). Due to varying results from the current experiment and previous studies, it is possible that the liver is not the most reliable biomarker of Mn status. According to Kincaid (2000), Mn taken up by the liver is excreted endogenously via the bile, and the accumulation of Mn in the liver often does not reflect dietary intakes of Mn. Underwood and Suttle (1999) also reported that liver Mn concentration does not respond to Mn supplementation, even at potentially toxic concentrations.

There was no treatment × day interaction (*P* = 0.45) or treatment effect (*P* = 0.82) for serum concentrations of Mn as shown in Figure 4b. There was an effect of day on Mn serum concentrations (*P* < 0.0001). Serum Mn concentrations decreased from d 0 through d 28 and then stabilized through harvest. Legleiter et al. (2005) reported that plasma Mn concentrations at harvest were not affected by supplemental Mn concentrations (Legleiter et al., 2005).

**Figure 5.**
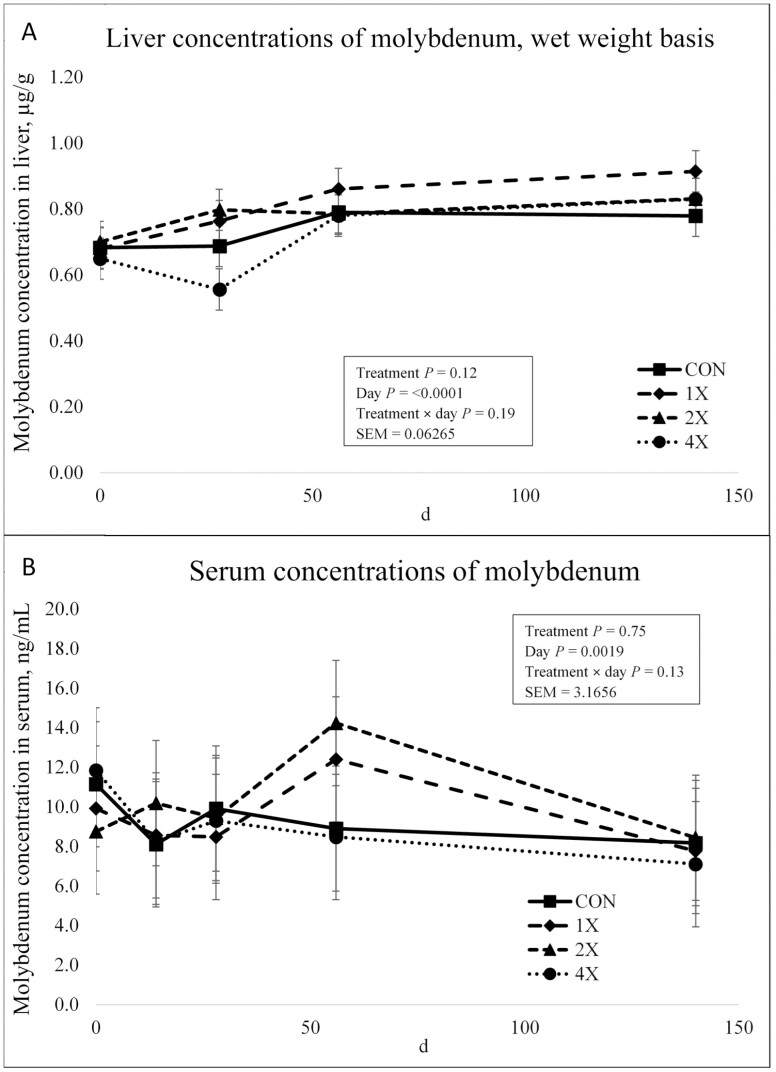
Liver (**a**) and serum (**b**) concentrations of molybdenum in finishing steers consuming 1 of 4 trace mineral supplements. Treatments included a control (**CON**; no supplemental trace minerals), **1X** (supplement containing 0.15 mg/kg of Co, 10 mg/kg of Cu, 50 mg/kg of Fe, 0.5 mg/kg of I, 20 mg/kg of Mn, 0.1 mg/kg of Se, and 30 mg/kg of Zn, on a DM basis), **2X** (supplement containing 0.30 mg/kg of Co, 20 mg/kg of Cu, 50 mg/kg of Fe, 1.0 mg/kg of I, 40 mg/kg of Mn, 0.2 mg/kg of Se, and 60 mg/kg of Zn, on a DM basis), or **4X** (supplement containing 0.60 mg/kg of Co, 40 mg/kg of Cu, 50 mg/kg of Fe, 2.0 mg/kg of I, 80 mg/kg of Mn, 0.3 mg/kg of Se, and 120 mg/kg of Zn on a DM basis). ^*^Cattle were harvested in 3 groups; d 126 (*n* =20 pens; 5 pens per treatment), d 140 (*n* = 16 pens; 4 pens per treatment), and d 154 (*n* = 12 pens; 3 pens per treatment). In this figure, 140 days on feed represents the final measurement, regardless of the actual harvest date. Values plotted represent least squares means ± SE of the mean, calculated for 12 animals per experimental group. Within time points, the slice output option of SAS (SAS Inst. Inc., Cary, NC) was used to perform mean separations.

There was no treatment × day interaction (*P* = 0.19) or effect of treatment (*P* = 0.12) on Mo concentrations in the liver as shown in Figure 5a. There was an effect of day on liver Mo concentrations (*P* < 0.0001). Liver Mo concentrations did not differ on d 0. However, Mo liver concentrations varied on d 28, tended to increase on d 56, and then plateaued through harvest. The present experiment did not provide supplemental Mo, so it was expected that regardless of treatment, Mo liver concentration would not differ. The changes in liver Mo concentrations over time could reflect use of Mo within the body. As Mo is an important antagonist for other TM, changes in Mo concentrations over time could influence the metabolism of other TM. Clawson et al. (1972) reported that the addition of supplemental Mo increased liver Mo concentrations.

There was no treatment × day interaction (*P* = 0.13) or effect of treatment (*P* = 0.75) for serum concentrations of Mo as shown in Figure 5b. There was an effect of day on serum Mo concentrations (*P* = 0.0019). Serum Mo concentrations decreased from d 0 through d 14. Serum Mo concentrations slightly increased on d 28 and then stabilized through harvest. To the authors knowledge, there are no published studies evaluating TM supplementation on serum Mo. However, Clawson et al. (1972) evaluated various Mo supplementation strategies on Mo plasma concentrations. Molybdenum plasma concentrations increased with the presence of supplemental Mo. Since Mo was not supplemented in this experiment, it was expected that there would be no effect of treatment on Mo serum concentrations.

**Figure 6.**
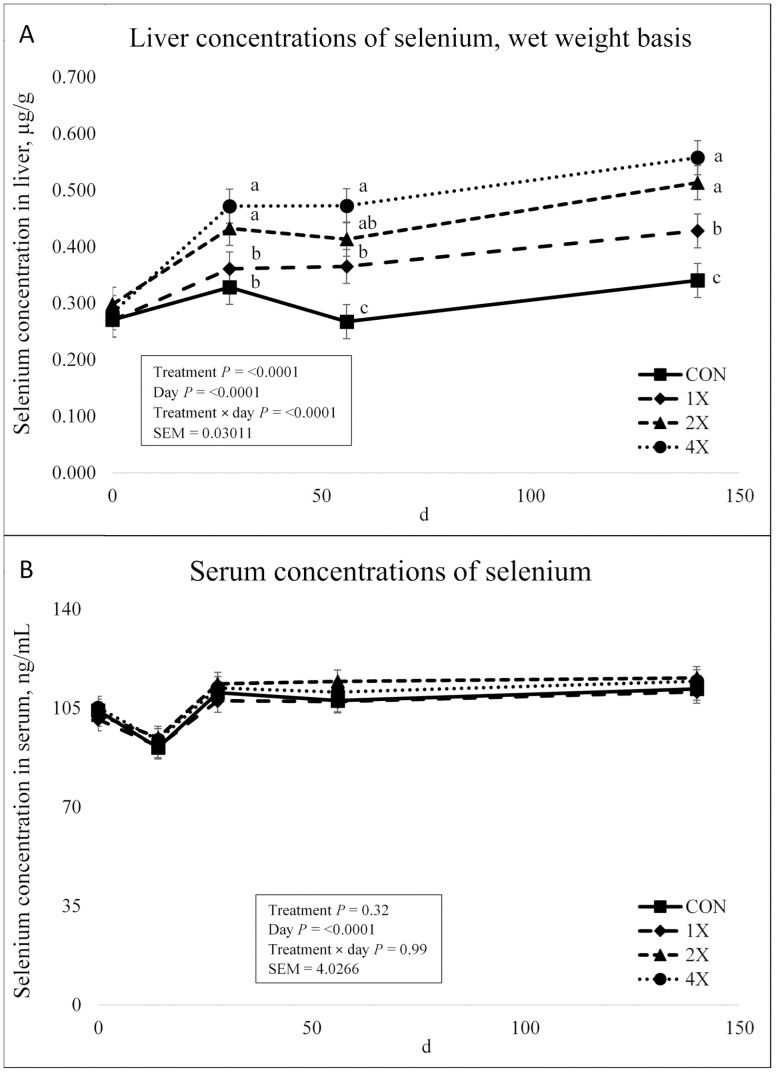
Liver (**a**) and serum (**b**) concentrations of selenium in finishing steers consuming 1 of 4 trace mineral supplements. Treatments included a control (**CON**; no supplemental trace minerals), **1X** (supplement containing 0.15 mg/kg of Co, 10 mg/kg of Cu, 50 mg/kg of Fe, 0.5 mg/kg of I, 20 mg/kg of Mn, 0.1 mg/kg of Se, and 30 mg/kg of Zn, on a DM basis), **2X** (supplement containing 0.30 mg/kg of Co, 20 mg/kg of Cu, 50 mg/kg of Fe, 1.0 mg/kg of I, 40 mg/kg of Mn, 0.2 mg/kg of Se, and 60 mg/kg of Zn, on a DM basis), or **4X** (supplement containing 0.60 mg/kg of Co, 40 mg/kg of Cu, 50 mg/kg of Fe, 2.0 mg/kg of I, 80 mg/kg of Mn, 0.3 mg/kg of Se, and 120 mg/kg of Zn on a DM basis). ^*^Cattle were harvested in 3 groups; d 126 (*n* =20 pens; 5 pens per treatment), d 140 (*n* = 16 pens; 4 pens per treatment), and d 154 (*n* = 12 pens; 3 pens per treatment). In this figure, 140 days on feed represents the final measurement, regardless of the actual harvest date.^a,b,c^Means within grouping without a common superscript letter differ (*P* < 0.05). Values plotted represent least squares means ± SE of the mean, calculated for 12 animals per experimental group. Within time points, the slice output option of SAS (SAS Inst. Inc., Cary, NC) was used to perform mean separations.

There was a treatment × day interaction for Se concentrations in the liver (*P* < 0.0001) as shown in Figure 6a. There was no difference in concentrations on d 0 among treatments. By d 28, all treatments displayed an increase in Se liver concentrations. Liver Se concentrations of 2X and 4X treatments did not differ from d 28 through harvest (*P* ≥ 0.11). However, liver Se concentrations of cattle on the 2X and 4X treatments were greater (*P* ≤ 0.05) on d 28 than cattle on the CON and 1X treatments, which did not differ (*P* = 0.37). Liver Se concentrations of 2X cattle did not differ from 1X cattle on d 56 (*P* = 0.19). Cattle offered the 1X, 2X, and 4X diets had greater liver Se concentrations than CON cattle on d 56 and at harvest (*P* ≤ 0.05). Arthington (2008) investigated source of Se against a negative control. Liver concentrations were greater in Se supplemented steers over the course of the experiment compared with nonsupplemented steers regardless of Se source. Niedermayer et al. (2018) reported TM supplementation increased liver Se concentrations. Steers supplemented with TM at the surveyed consulting nutritionist mode (Samuelson et al., 2016) had greater liver Se than control steers on d 70. Cattle supplemented with TM had greater Se liver concentrations at harvest (Niedermayer et al., 2018). Previous research indicates a similar response to Se supplementation, however, liver Se concentrations indicative of Se deficiency in cattle vary substantially within the literature. Liver Se concentrations within the current experiment were considered adequate for all experimental treatments (Puls, 1994; Thompson et al., 1998).

There was no treatment × day interaction (*P* = 0.99) or effect of treatment (*P* = 0.32) for Se concentrations in serum as shown in Figure 6b. There was an effect of day on serum Se concentrations (*P* < 0.0001). Serum Se concentrations exhibited a decrease from d 0 through d 14 and then an increase on d 28. Serum Se concentrations remained stable through harvest. In classical research in sheep, serum Se increased as dietary Se increased (Henry et al., 1988). In general, Perry et al. (1976) reported that Se concentrations in serum increased with supplementation and over time; however, statistical analysis was not performed due to the nature of sampling. Based on liver and serum Se concentrations in this experiment, liver Se concentrations appear to be more indicative of the effects Se supplementation on Se status in cattle.

**Figure 7.**
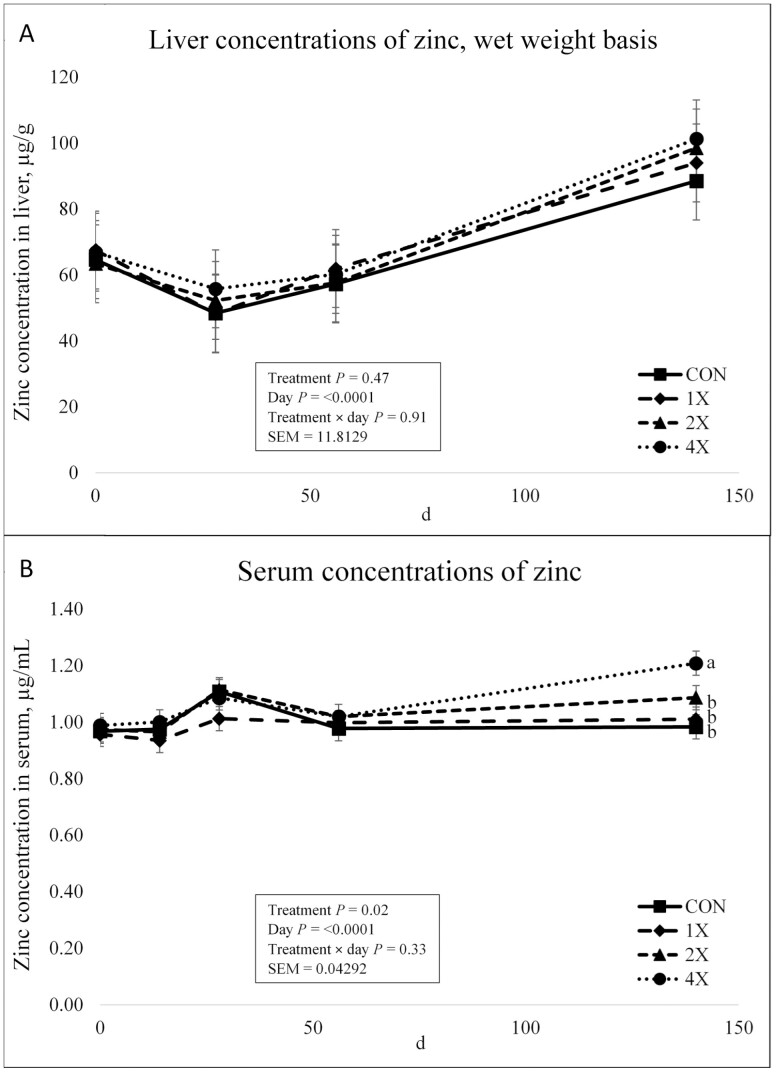
Liver (**a**) and serum (**b**) concentrations of zinc in finishing steers consuming 1 of 4 trace mineral supplements. Treatments included a control (**CON**; no supplemental trace minerals), **1X** (supplement containing 0.15 mg/kg of Co, 10 mg/kg of Cu, 50 mg/kg of Fe, 0.5 mg/kg of I, 20 mg/kg of Mn, 0.1 mg/kg of Se, and 30 mg/kg of Zn, on a DM basis), **2X** (supplement containing 0.30 mg/kg of Co, 20 mg/kg of Cu, 50 mg/kg of Fe, 1.0 mg/kg of I, 40 mg/kg of Mn, 0.2 mg/kg of Se, and 60 mg/kg of Zn, on a DM basis), or **4X** (supplement containing 0.60 mg/kg of Co, 40 mg/kg of Cu, 50 mg/kg of Fe, 2.0 mg/kg of I, 80 mg/kg of Mn, 0.3 mg/kg of Se, and 120 mg/kg of Zn on a DM basis). ^*^Cattle were harvested in 3 groups; d 126 (*n* =20 pens; 5 pens per treatment), d 140 (*n* = 16 pens; 4 pens per treatment), and d 154 (*n* = 12 pens; 3 pens per treatment). In this figure, 140 days on feed represents the final measurement, regardless of the actual harvest date. ^a,b,c^Means within grouping without a common superscript letter differ (*P* < 0.05). Values plotted represent least squares means ± SE of the mean, calculated for 12 animals per experimental group. Within time points, the slice output option of SAS (SAS Inst. Inc., Cary, NC) was used to perform mean separations.

There was no treatment × day interaction (*P* = 0.91) or effect of treatment (*P* = 0.47) for Zn concentrations in the liver as shown in Figure 7a. However, there was an effect of day on Zn concentrations (*P* < 0.0001). Liver Zn concentrations decreased from d 0 through d 28. Liver Zn concentrations then increased for all treatments through d 56 and continued to increase through harvest. Berrett et al. (2015) reported that TM supplementation had no effect on Zn liver concentrations at harvest. Niedermayer et al. (2018) reported Zn liver concentrations were not affected by TM supplementation on d 70. However, steers receiving TM supplementation at requirement levels had greater liver Zn concentrations than control cattle and steers supplemented at consulting nutritionist modes at harvest (Niedermayer et al., 2018).

There was no treatment × day interaction for serum Zn concentrations (*P* = 0.33). However, there was an effect of treatment (*P* = 0.02) and day (*P* < 0.0001) on serum Zn concentrations as shown in Figure 7b. Serum Zn concentrations did not differ on d 0 through d 14. On d 28, all cattle displayed a slight increase in concentrations followed by a slight decrease in Zn concentrations on d 56. Serum Zn concentrations for cattle on the 4X treatment were greater at harvest than cattle on the CON, 1X, and 2X treatments (*P* = 0.001). Greene et al. (1988) reported Zn supplementation and source did not affect Zn serum concentrations in feedlot steers. It is well accepted that plasma Zn is not a reliable indicator of Zn status unless animals are severely deficient in Zn (Underwood and Suttle, 1999). It is also probable that serum Zn concentrations are not the most accurate measure of Zn status within an animal.

### Antibody Concentrations

**Figure 8.**
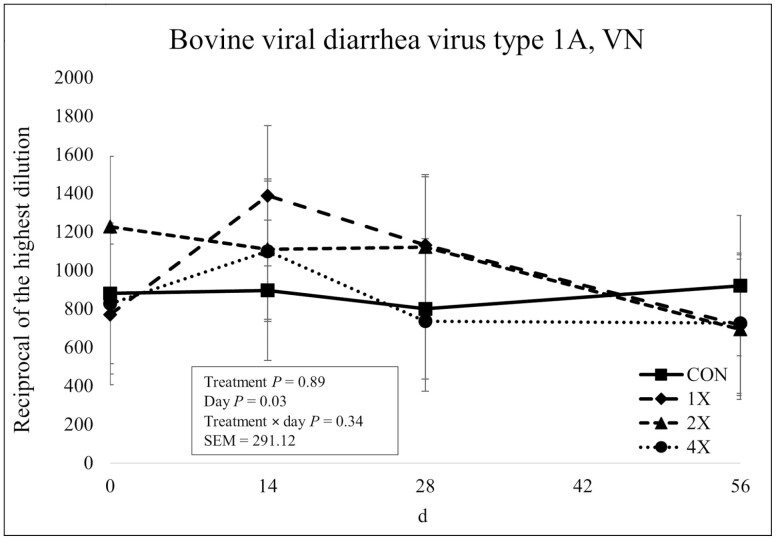
Virus neutralization of bovine viral diarrhea virus type 1A in finishing steers consuming 1 of 4 trace mineral supplements. Treatments included a control (**CON**; no supplemental trace minerals), **1X** (supplement containing 0.15 mg/kg of Co, 10 mg/kg of Cu, 50 mg/kg of Fe, 0.5 mg/kg of I, 20 mg/kg of Mn, 0.1 mg/kg of Se, and 30 mg/kg of Zn, on a DM basis), **2X** (supplement containing 0.30 mg/kg of Co, 20 mg/kg of Cu, 50 mg/kg of Fe, 1.0 mg/kg of I, 40 mg/kg of Mn, 0.2 mg/kg of Se, and 60 mg/kg of Zn, on a DM basis), or **4X** (supplement containing 0.60 mg/kg of Co, 40 mg/kg of Cu, 50 mg/kg of Fe, 2.0 mg/kg of I, 80 mg/kg of Mn, 0.3 mg/kg of Se, and 120 mg/kg of Zn on a DM basis). The virus neutralization (VN; or serum neutralization, SN) antibody titer is reported as the reciprocal of the highest dilution of serum that neutralizes the infectivity of the virus (e.g., endpoint dilution 1:128 = antibody titer of 128). Values reported with a less than symbol (<) indicate no detectable antibody at the lowest readable dilution (e.g., <4 = no detectable antibody at a 1:4 dilution). Values reported with a greater than symbol (>) indicate titers that are greater than or equal to the highest dilution of the test (e.g., >16 = presence of antibody greater than or equal to a dilution of 1:16). Samples with an endpoint dilution greater than 4,096 are reported as 4,096. Values plotted represent least squares means ± SE of the mean, calculated for 12 animals per experimental group. Within time points, the slice output option of SAS (SAS Inst. Inc., Cary, NC) was used to perform mean separations.

**Figure 9.**
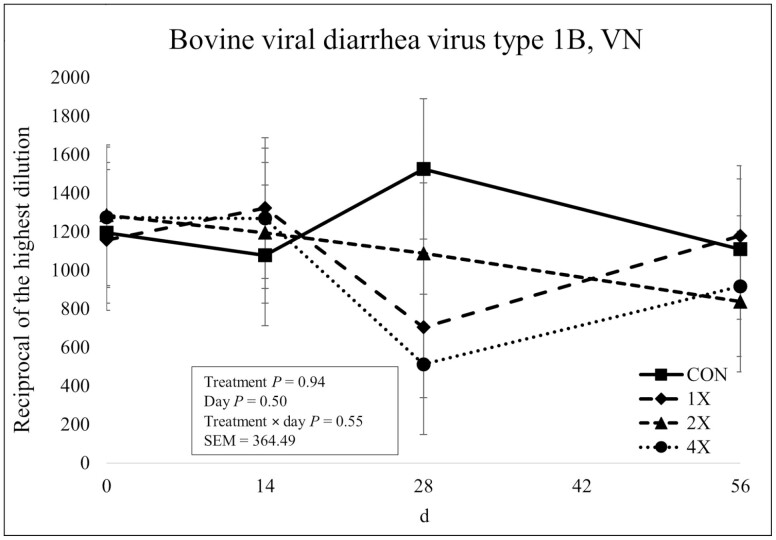
Virus neutralization of bovine viral diarrhea virus Type 1B in finishing steers consuming 1 of 4 trace mineral supplements. Treatments included a control (**CON**; no supplemental trace minerals), **1X** (supplement containing 0.15 mg/kg of Co, 10 mg/kg of Cu, 50 mg/kg of Fe, 0.5 mg/kg of I, 20 mg/kg of Mn, 0.1 mg/kg of Se, and 30 mg/kg of Zn, on a DM basis), **2X** (supplement containing 0.30 mg/kg of Co, 20 mg/kg of Cu, 50 mg/kg of Fe, 1.0 mg/kg of I, 40 mg/kg of Mn, 0.2 mg/kg of Se, and 60 mg/kg of Zn, on a DM basis), or **4X** (supplement containing 0.60 mg/kg of Co, 40 mg/kg of Cu, 50 mg/kg of Fe, 2.0 mg/kg of I, 80 mg/kg of Mn, 0.3 mg/kg of Se, and 120 mg/kg of Zn on a DM basis). The virus neutralization (VN; or serum neutralization, SN) antibody titer is reported as the reciprocal of the highest dilution of serum that neutralizes the infectivity of the virus (e.g., endpoint dilution 1:128 = antibody titer of 128). Values reported with a less than symbol (<) indicate no detectable antibody at the lowest readable dilution (e.g., <4 = no detectable antibody at a 1:4 dilution). Values reported with a greater than symbol (>) indicate titers that are greater than or equal to the highest dilution of the test (e.g., >16 = presence of antibody greater than or equal to a dilution of 1:16). Samples with an endpoint dilution greater than 4,096 are reported as 4,096. Values plotted represent least squares means ± SE of the mean, calculated for 12 animals per experimental group. Within time points, the slice output option of SAS (SAS Inst. Inc., Cary, NC) was used to perform mean separations.

**Figure 10.**
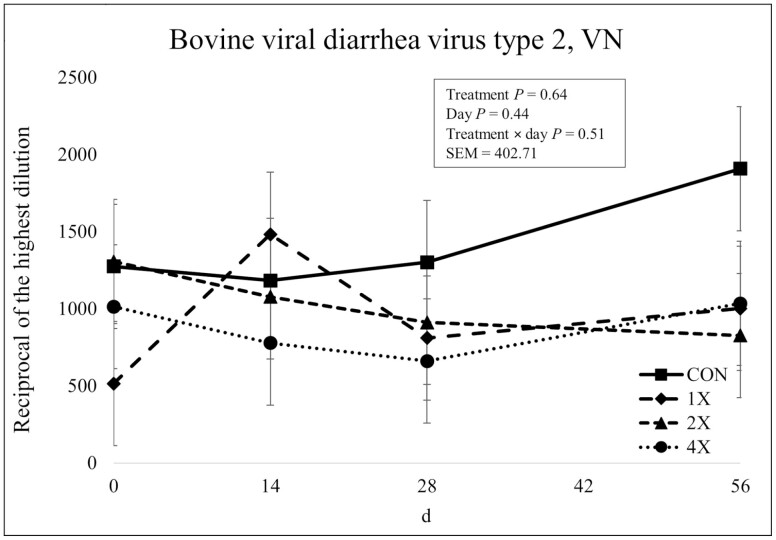
Virus neutralization of bovine viral diarrhea virus Type 2 in finishing steers consuming 1 of 4 trace mineral supplements. Treatments included a control (**CON**; no supplemental trace minerals), **1X** (supplement containing 0.15 mg/kg of Co, 10 mg/kg of Cu, 50 mg/kg of Fe, 0.5 mg/kg of I, 20 mg/kg of Mn, 0.1 mg/kg of Se, and 30 mg/kg of Zn, on a DM basis), **2X** (supplement containing 0.30 mg/kg of Co, 20 mg/kg of Cu, 50 mg/kg of Fe, 1.0 mg/kg of I, 40 mg/kg of Mn, 0.2 mg/kg of Se, and 60 mg/kg of Zn, on a DM basis), or **4X** (supplement containing 0.60 mg/kg of Co, 40 mg/kg of Cu, 50 mg/kg of Fe, 2.0 mg/kg of I, 80 mg/kg of Mn, 0.3 mg/kg of Se, and 120 mg/kg of Zn on a DM basis). The virus neutralization (VN; or serum neutralization, SN) antibody titer is reported as the reciprocal of the highest dilution of serum that neutralizes the infectivity of the virus (e.g., endpoint dilution 1:128 = antibody titer of 128). Values reported with a less than symbol (<) indicate no detectable antibody at the lowest readable dilution (e.g., <4 = no detectable antibody at a 1:4 dilution). Values reported with a greater than symbol (>) indicate titers that are greater than or equal to the highest dilution of the test (e.g., >16 = presence of antibody greater than or equal to a dilution of 1:16). Samples with an endpoint dilution greater than 4,096 are reported as 4,096. Values plotted represent least squares means ± SE of the mean, calculated for 12 animals per experimental group. Within time points, the slice output option of SAS (SAS Inst. Inc., Cary, NC) was used to perform mean separations.

There was no treatment × day interaction (*P* = 0.34) or effect of treatment (*P* = 0.89) for BVDV Type 1A antibody concentrations as shown in Figure 8. However, there was an effect of day on BVDV Type 1A antibody concentrations (*P* = 0.03). Antibody concentrations for BVDV Type 1A increased from d 0 to d 14 then gradually decreased over the remainder of the sampling period. There was no treatment × day interaction, effect of treatment, or effect of day on BVDV Type 1B or BVDV Type 2 antibody concentrations (*P* ≥ 0.44) as shown in Figures 9 and 10. Previous research involving injectable TM has reported only a tendency (Roberts et al., 2016) or no treatment × day interaction for neutralizing antibody concentrations for BVDV Type 1 and BVDV Type 2 for cattle injected with supplemental TM (Arthington and Havenga, 2012).

**Figure 11.**
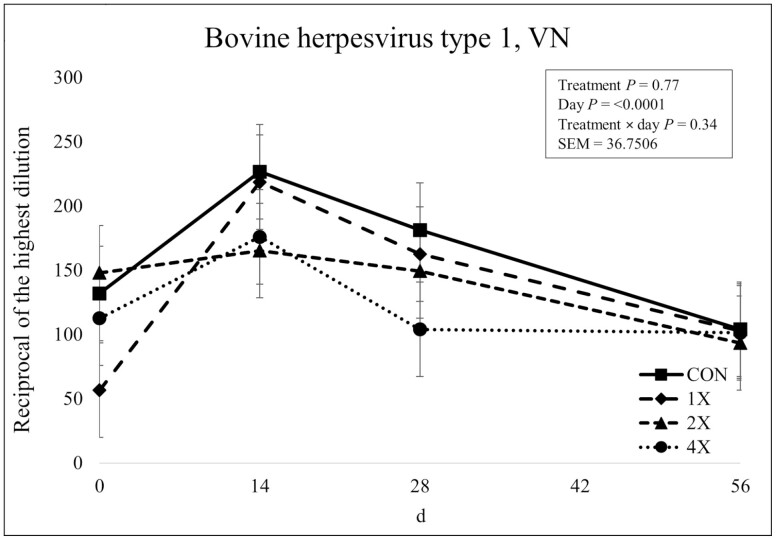
Virus neutralization of bovine herpesvirus type 1 in finishing steers consuming 1 of 4 trace mineral supplements. Treatments included a control (**CON**; no supplemental trace minerals), **1X** (supplement containing 0.15 mg/kg of Co, 10 mg/kg of Cu, 50 mg/kg of Fe, 0.5 mg/kg of I, 20 mg/kg of Mn, 0.1 mg/kg of Se, and 30 mg/kg of Zn, on a DM basis), **2X** (supplement containing 0.30 mg/kg of Co, 20 mg/kg of Cu, 50 mg/kg of Fe, 1.0 mg/kg of I, 40 mg/kg of Mn, 0.2 mg/kg of Se, and 60 mg/kg of Zn, on a DM basis), or **4X** (supplement containing 0.60 mg/kg of Co, 40 mg/kg of Cu, 50 mg/kg of Fe, 2.0 mg/kg of I, 80 mg/kg of Mn, 0.3 mg/kg of Se, and 120 mg/kg of Zn on a DM basis). The virus neutralization (VN; or serum neutralization, SN) antibody titer is reported as the reciprocal of the highest dilution of serum that neutralizes the infectivity of the virus (e.g., endpoint dilution 1:128 = antibody titer of 128). Values reported with a less than symbol (<) indicate no detectable antibody at the lowest readable dilution (e.g., <4 = no detectable antibody at a 1:4 dilution). Values reported with a greater than symbol (>) indicate titers that are greater than or equal to the highest dilution of the test (e.g., >16 = presence of antibody greater than or equal to a dilution of 1:16). Samples with an endpoint dilution greater than 4,096 are reported as 4,096. Values plotted represent least squares means ± SE of the mean, calculated for 12 animals per experimental group. Within time points, the slice output option of SAS (SAS Inst. Inc., Cary, NC) was used to perform mean separations.

There was no treatment × day interaction (*P* = 0.34) or effect of treatment (*P* = 0.77) for BHV-1 concentrations. However, there was an effect of day (*P* < 0.0001) as shown in Figure 11. Antibody concentrations for BHV-1 increased from d 0 to d 14, then gradually decreased over the remainder of the sampling period. Rhoads et al. (2003) reported that TM supplementation had no effect on antibody concentrations specific to IBR. However, Arthington and Havenga (2012) reported that an injectable TM supplement containing Cu, Mn, Zn, and Se increased neutralizing antibody concentrations against BHV-1 on d 14, 30, and 60 postvaccination compared with a control. It should be noted that cattle used by Arthington and Havenga (2012) had no previous vaccination exposure. It is plausible that an increase was not elicited in the current experiment due to previous vaccination at arrival combined with unknown vaccination status prior to arrival. It is also plausible that injectable TM are more influential on the vaccine induced antibody response, due to the rapid availability compared to dietary TM supplementation.

**Figure 12.**
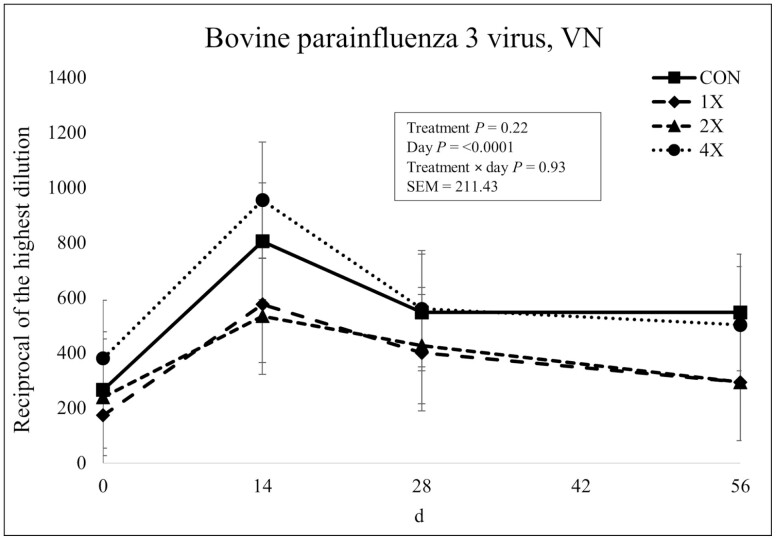
Virus neutralization of bovine parainfluenza 3 virus in finishing steers consuming 1 of 4 trace mineral supplements. Treatments included a control (**CON**; no supplemental trace minerals), **1X** (supplement containing 0.15 mg/kg of Co, 10 mg/kg of Cu, 50 mg/kg of Fe, 0.5 mg/kg of I, 20 mg/kg of Mn, 0.1 mg/kg of Se, and 30 mg/kg of Zn, on a DM basis), **2X** (supplement containing 0.30 mg/kg of Co, 20 mg/kg of Cu, 50 mg/kg of Fe, 1.0 mg/kg of I, 40 mg/kg of Mn, 0.2 mg/kg of Se, and 60 mg/kg of Zn, on a DM basis), or **4X** (supplement containing 0.60 mg/kg of Co, 40 mg/kg of Cu, 50 mg/kg of Fe, 2.0 mg/kg of I, 80 mg/kg of Mn, 0.3 mg/kg of Se, and 120 mg/kg of Zn on a DM basis). The virus neutralization (VN; or serum neutralization, SN) antibody titer is reported as the reciprocal of the highest dilution of serum that neutralizes the infectivity of the virus (e.g., endpoint dilution 1:128 = antibody titer of 128). Values reported with a less than symbol (<) indicate no detectable antibody at the lowest readable dilution (e.g., <4 = no detectable antibody at a 1:4 dilution). Values reported with a greater than symbol (>) indicate titers that are greater than or equal to the highest dilution of the test (e.g., >16 = presence of antibody greater than or equal to a dilution of 1:16). Samples with an endpoint dilution greater than 4,096 are reported as 4,096. Values plotted represent least squares means ± SE of the mean, calculated for 12 animals per experimental group. Within time points, the slice output option of SAS (SAS Inst. Inc., Cary, NC) was used to perform mean separations.

There was no treatment × day interaction (*P* = 0.93) or effect of treatment (*P* = 0.22) for PI-3 antibody concentrations as shown in Figure 12. However, there was an effect of day (*P* < 0.0001). Antibody concentrations were similar on d 0. Similar to BVDV Type 1A and BHV-1, antibody concentrations for PI-3 increased from d 0 to d 14, then gradually decreased over the remainder of the sampling period. George et al. (1997) evaluated heifers that had been vaccinated only with clostridial bacterins prior to arrival at a research feedlot. All heifers were vaccinated for viral respiratory pathogens upon arrival. Treatments consisted of a basal 55% concentrate receiving diet with supplemental inorganic or organic sources of Co, Cu, Mn, and Zn. Although calves had antibodies to PI-3 on arrival, the secondary PI-3 antibody response on d 14 and 28 after arrival vaccination was increased by organically complexed minerals. However, TM supplementation was not evaluated against a negative control (George et al., 1997).

Antibody concentration data from the current experiment and previous research report varying results. Cattle in the current experiment were considered low to moderate risk (Wilson et al., 2017). Vaccination status prior to arrival was unknown but cattle received respiratory vaccinations at arrival, and prior to allocation to treatments. Cattle were also older, heavier at arrival, and had the opportunity to acclimate to the feedlot environment prior to the initiation of experimental TM treatments. Many previous studies examining the effect of TM supplementation on antibody concentrations have been conducted using high-risk receiving calves.

## CONCLUSION

Trace mineral supplementation had no effect on overall cattle performance or carcass characteristics in the current experiment. Antibody concentrations for common respiratory viruses were also not affected by supplemental TM inclusion. The supplementation of TM did have an impact on serum Co concentrations, liver Cu concentrations, liver Se concentrations, and serum Zn concentrations, where increased dietary inclusion of TM increased the previously mentioned TM concentrations over time. There are several reasons why TM supplementation may not have effected overall cattle performance, carcass characteristics, and antibody titers in the current experiment. All cattle were receiving sufficient TM concentrations from the basal diet, with the exception of Cu, during the 42-d depletion period without additional TM supplementation. As such, it is probable that additional supplementation would not enhance performance or carcass characteristics, due to the presence of sufficient levels of most TM in the basal diet. It should also be noted that cattle were considered to be low to moderate risk upon arrival and were acclimated to the feedlot environment during the depletion period prior to the allocation of dietary treatments. As such, there was not an initial depression in DMI that could result in the potential for decreased TM consumption from the basal diet at the beginning of the experiment. Based on the overall performance, carcass characteristics, and antibody concentrations observed in this experiment, the supplementation of TM beyond the NASEM (2016) recommended requirements for the entire finishing period does not appear to be warranted. However, this experiment does indicate that increased supplementation of TM while feeding a beta agonist immediately prior to harvest may result in synergistic effects. While these effects did not influence overall performance or carcass traits in the current experiment, increased TM supplementation immediately prior to and during the beta agonist feeding period could be of value to the feedlot industry and should be investigated further. However, if nutritionists do not take into consideration the concentrations of TM from the basal diet when making TM supplementation recommendations, as indicated by survey data, then the additional supplementation of TM at published requirement levels (NASEM, 2016) appears to be sufficient to optimize animal performance and carcass characteristics even when feeding a beta agonist.
