# Correction to: The effects of varying levels of trace mineral supplementation on performance, carcass characteristics, mineral balance, and antibody concentrations in feedlot cattle

**DOI:** 10.1093/tas/txac137

**Published:** 2022-10-12

This is a correction to: Brittany A. Lippy, Colton A. Robison, Blake K. Wilson, The effects of varying levels of trace mineral supplementation on performance, carcass characteristics, mineral balance, and antibody concentrations in feedlot cattle, *Translational Animal Science*, Volume 6, Issue 3, July 2022, txac093, https://doi.org/10.1093/tas/txac093. In the originally published version of this manuscript, there was an error in how the liver minerals concentrations were referenced. The levels were reported on a wet weight basis while the references used for discussing normal/reference ranges within the manuscript were reported on a dry basis. The authors mistakenly believed their results were also reported on dry basis. This has been corrected online.

